# Omega-3 Fatty Acids Mitigate Long-Lasting Disruption of the Endocannabinoid System in the Adult Mouse Hippocampus Following Adolescent Binge Drinking

**DOI:** 10.3390/ijms26125507

**Published:** 2025-06-09

**Authors:** Maitane Serrano, Miquel Saumell-Esnaola, Garazi Ocerin, Gontzal García del Caño, Edgar Soria-Gómez, Amaia Mimenza, Nagore Puente, Itziar Bonilla-Del Río, Almudena Ramos-Uriarte, Leire Reguero, Brian R. Christie, Fernando Rodríguez de Fonseca, Marta Rodríguez-Arias, Inmaculada Gerrikagoitia, Pedro Grandes

**Affiliations:** 1 Department of Neurosciences, Faculty of Medicine and Nursing, University of the Basque Country UPV/EHU, 48940 Leioa, Spain; maitane.serrano@ehu.eus (M.S.); garazi.ocerin@ehu.eus (G.O.); edgar.soria@achucarro.org (E.S.-G.); amaia.mimenza@ehu.eus (A.M.); nagore.puente@ehu.eus (N.P.); itziar.bonilla@ehu.eus (I.B.-D.R.); almudena.ramos@ehu.eus (A.R.-U.); leire.reguero@ehu.eus (L.R.); 2 Achucarro Basque Center for Neuroscience, Science Park of the UPV/EHU, 48940 Leioa, Spain; 3 Department of Pharmacology, Faculty of Pharmacy, University of the Basque Country UPV/EHU, 01006 Vitoria-Gasteiz, Spain; miquel.saumell@ehu.eus; 4 Bioaraba, Neurofarmacología Celular y Molecular, 01006 Vitoria-Gasteiz, Spain; gontzal.garcia@ehu.eus; 5 Department of Neurosciences, Faculty of Pharmacy, University of the Basque Country UPV/EHU, 01006 Vitoria-Gasteiz, Spain; 6 Red de Investigación en Atención Primaria de Adicciones (RIAPAD), ISCIII, 28029 Madrid, Spain; fernando.rodriguez@ibima.eu (F.R.d.F.); marta.rodriguez@uv.es (M.R.-A.); 7 Ikerbasque, Basque Foundation for Science, 48009 Bilbao, Spain; 8 Division of Medical Sciences and Institute for Aging and Lifelong Health, University of Victoria, Victoria, BC V8P 5C2, Canada; brain64@uvic.ca; 9 Island Medical Program, Cellular and Physiological Sciences, Djavad Mowafaghian Centre for Brain Health, University of British Columbia, Vancouver, BC V6T 1Z3, Canada; 10 Center for Behavioral Teratology, San Diego State University, San Diego, CA 92182, USA; 11 Mental Health Clinical Management Unit, Institute of Biomedical Research of Málaga-IBIMA, Regional University Hospital of Málaga, 29590 Málaga, Spain; 12 Department of Psychobiology, Faculty of Psychology, Universitat de València, 46010 Valencia, Spain

**Keywords:** alcohol, CB1 receptor, hippocampus, memory, polyunsaturated fatty acids, synaptic plasticity

## Abstract

Adolescent binge drinking has lasting behavioral consequences by disrupting the endocannabinoid system (ECS) and depleting brain omega-3. The natural accumulation of omega-3 fatty acids in cell membranes is crucial for maintaining the membrane structure, supporting interactions with the ECS, and restoring synaptic plasticity and cognition impaired by prenatal ethanol (EtOH) exposure. However, it remains unclear whether omega-3 supplementation can mitigate the long-term effects on the ECS, endocannabinoid-dependent synaptic plasticity, and cognition following adolescent binge drinking. Here, we demonstrated that omega-3 supplementation during EtOH withdrawal increases CB1 receptors in hippocampal presynaptic terminals of male mice, along with the recovery of receptor-stimulated [^35^S]GTPγS binding to Gαi/o proteins. These changes are associated with long-term potentiation (LTP) at excitatory medial perforant path (MPP) synapses in the dentate gyrus (DG), which depends on anandamide (AEA), transient receptor potential vanilloid 1 (TRPV1), and N-methyl-D-aspartate (NMDA) receptors. Finally, omega-3 intake following binge drinking reduced the time and number of errors required to locate the escape box in the Barnes maze test. Collectively, these findings suggest that omega-3 supplementation restores Barnes maze performance to levels comparable to those of control mice after adolescent binge drinking. This recovery is likely mediated by modulation of the hippocampal ECS, enhancing endocannabinoid-dependent excitatory synaptic plasticity.

## 1. Introduction

Adolescent binge drinking has significant consequences for brain development as it disrupts synaptic transmission and neuroplasticity during a critical period of structural and functional maturation. These disruptions can lead to cognitive, emotional, and motor impairments [1,2]. A growing body of evidence highlights the involvement of the ECS in mediating the acute, chronic, and withdrawal effects of EtOH on synaptic function, as well as in the negative outcomes associated with AUD [3,4,5,6]. Furthermore, numerous studies emphasize the role of the ECS in modulating behavioral responses to both EtOH exposure and withdrawal [7].

The ECS comprises several key components: G protein-coupled cannabinoid receptors (primarily CB1 and CB2); endocannabinoids produced on demand—mainly 2-arachidonoylglycerol (2-AG) and AEA; and the enzymes responsible for their synthesis (diacylglycerol lipase-α [DAGLα] for 2-AG and N-acyl phosphatidylethanolamine-specific phospholipase D [NAPE-PLD] for AEA), degradation (monoacylglycerol lipase [MAGL] for 2-AG and fatty acid amide hydrolase [FAAH] for AEA), and transport [8].

Inhibition of 2-AG synthesis has been shown to reduce EtOH consumption in mouse models of AUD [9,10], and drinking behavior has been closely linked to changes in CB1 receptor expression and activity [11]. Accordingly, voluntary EtOH intake was significantly reduced in CB1 knockout mice, which also failed to exhibit dopamine release in the nucleus accumbens in response to EtOH [12]. Furthermore, reducing CB1 receptor activity using antagonist compounds has been shown to alter EtOH drinking behavior [13].

Chronic EtOH exposure also decreases CB1 receptor mRNA levels, thereby altering receptor expression and function. We have previously shown that adolescent binge drinking reduces CB1 mRNA expression in the mature hippocampus, ultimately leading to decreased CB1 receptor levels at excitatory terminals in the hippocampal DG. These alterations, along with increased MAGL mRNA and impaired endocannabinoid (eCB)-dependent long-term depression (LTD), contribute to memory deficits [3,14]. Notably, increasing 2-AG levels through inhibition of MAGL enzymatic activity can restore the synaptic plasticity and cognitive function deficits induced by adolescent binge drinking [3].

While pharmacological approaches offer potential strategies to address EtOH-induced cognitive deficits, nutritional supplementation with dietary omega-3 fatty acids may also provide practical therapeutic benefits. In this context, adequate intake of omega-3 polyunsaturated fatty acids (PUFAs)—sourced from cold-water fatty fish, nuts, seeds, and plant oils—and ideally maintaining an omega-6/omega-3 ratio of 2:1 to 5:1, is essential for optimal physiological homeostasis. However, dietary patterns, particularly in Western countries, have shifted in recent decades, resulting in an imbalanced omega-6/omega-3 ratio of approximately 20–30:1 [15,16].

Omega-3 intake enhances hippocampal synaptic transmission and supports hippocampal-dependent memory functions [17], whereas EtOH consumption depletes omega-3 levels through multiple mechanisms [18,19]. Docosahexaenoic acid (DHA, 22:6) and eicosapentaenoic acid (EPA, 20:5), two key omega-3 PUFAs that accumulate in brain cell membranes, help preserve membrane structure and fluidity. These fatty acids can cross the blood–brain barrier and persist in the brain for over a month. They restore glutathione levels, reduce oxidative stress and apoptosis, exhibit anti-inflammatory properties, and counteract synaptic plasticity deficits—thereby mitigating cognitive impairments caused by prenatal EtOH exposure [20,21,22]. Due to their prolonged presence in the central nervous system (CNS), DHA and EPA are of particular interest in neurobiological research [23].

The negative impact of EtOH on DHA disrupts synaptic plasticity in the hippocampus and medial prefrontal cortex—regions that are naturally enriched in DHA [22]. Enhancing endocannabinoid signaling has been shown to restore emotional and cognitive functions, as well as reverse eCB-dependent synaptic plasticity deficits caused by omega-3 deficiency in brain regions involved in mood and cognition [24,25]. In addition, omega-3 supplementation in adult mice has been shown to restore CB1 receptor expression disrupted by adolescent EtOH exposure in brain regions associated with motor and cognitive functions [26].

Despite the well-established link between EtOH, omega-3 fatty acids, and the ECS, the specific effects of omega-3 PUFA supplementation on the expression and function of ECS components following adolescent EtOH exposure remain largely unclear. Further research is needed to enhance the efficacy of omega-3 PUFAs in reducing EtOH-induced brain damage and the associated cognitive and behavioral impairments [23].

This study investigated the potential of dietary supplementation with 1.2% EPA and 0.8% DHA, focusing on the mode of administration and timing of exposure. It specifically examined how this intervention mitigates the long-term adverse effects of adolescent binge drinking on synaptic transmission, CB1 receptor-dependent synaptic plasticity, and cognitive function.

## 2. Results

### 2.1. Immunolocalization of CB1 Receptors in DG Neurons and Astrocytes

**Figure 1:**
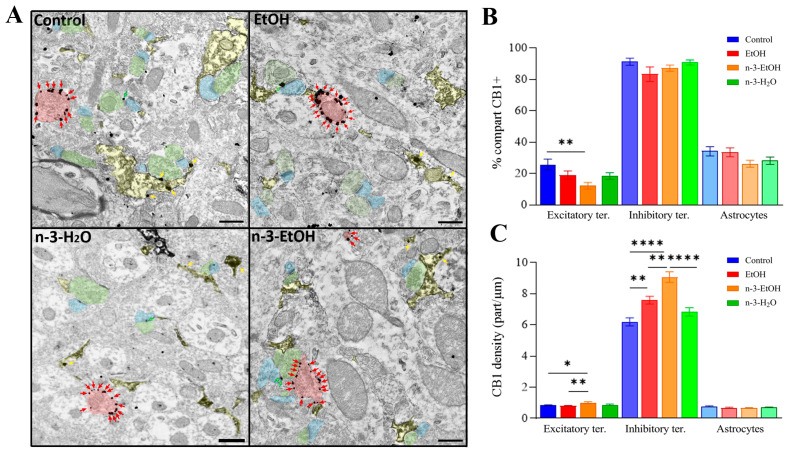
Immunoelectron localization of CB1 receptors in the middle third of the DG in control, EtOH, n-3-EtOH and n-3-H_2_O mice. (**A**) CB1 receptors were identified in terminals forming symmetric synapses (red shading and arrows), terminals forming asymmetric synapses (green shading and arrows) with dendrites (blue shading) and astrocytic membranes (yellow shading and arrows). (**B**) Percentage of CB1 receptor-positive profiles (excitatory terminals: n-3-EtOH vs. control *p* = 0.0037). (**C**) CB1 receptor density (particles/µm) in excitatory terminals (n-3-EtOH vs. control *p* = 0.0132; n-3-EtOH vs. EtOH *p* = 0.0015), inhibitory terminals (EtOH vs. control *p* = 0.0031; n-3-EtOH vs. control *p* < 0.0001; n-3-EtOH vs. EtOH *p* = 0.0012; n-3-H_2_O vs. n-3-EtOH *p* < 0.0001), and GLAST-stained astrocytes. Results are presented as means ± SEMs (see Supplementary Table S1). Statistical analysis of data used two-way ANOVA with Tukey’s multiple comparison tests: * *p* < 0.05, ** *p* < 0.01, **** *p* < 0.0001. Scale bars: 200 nm.

The pre-embedding immunogold technique was used for immunolocalizing CB1 receptors in the DG by electron microscopy. CB1-immunopositive gold particles were observed in both excitatory and inhibitory presynaptic terminals, forming asymmetric and symmetric synapses with dendritic spines and dendrites, respectively (Figure 1A). The percentage of excitatory terminals containing CB1 receptor particles remained virtually unchanged among the control, EtOH, and n-3-H_2_O groups, but was reduced in the n-3-EtOH group (Figure 1B; Supplementary Table S1). The percentage of inhibitory terminals labeled with CB1 receptors did not differ significantly among the experimental groups, and, as expected, was considerably higher than the proportion of labeled excitatory terminals (Figure 1B; Supplementary Table S1).

The density of CB1 receptor particles was markedly higher at inhibitory terminals than at excitatory terminals, consistent with findings widely reported in the literature. In excitatory terminals, no significant differences in CB1 receptor density were observed between the control and EtOH groups. However, a significantly higher density was found in the n-3-EtOH group compared to both groups maintained on a standard diet (Figure 1C; Supplementary Table S1). In contrast, the CB1 receptor density at inhibitory terminals differed significantly between groups. In particular, the density in the EtOH group was considerably higher and significantly different from that in the control group. Notably, omega-3 supplementation further increased CB1 receptor density following EtOH exposure compared to the EtOH group without supplementation (Figure 1C; Supplementary Table S1).

Finally, glutamate aspartate transporter (GLAST) immunohistochemical staining combined with pre-embedding immunogold labeling for the CB1 receptor was used to identify CB1-positive astrocytic processes [27]. The percentage of CB1-positive, GLAST-stained astrocytic processes, as well as the CB1 receptor density in this compartment, were similar across experimental groups, with no statistically significant differences observed (Figure 1A–C; Supplementary Table S1).

### 2.2. Expression and CB1 Receptor Coupling to Gα_i/o_ Proteins in Hippocampal Synaptosomes

**Figure 2:**
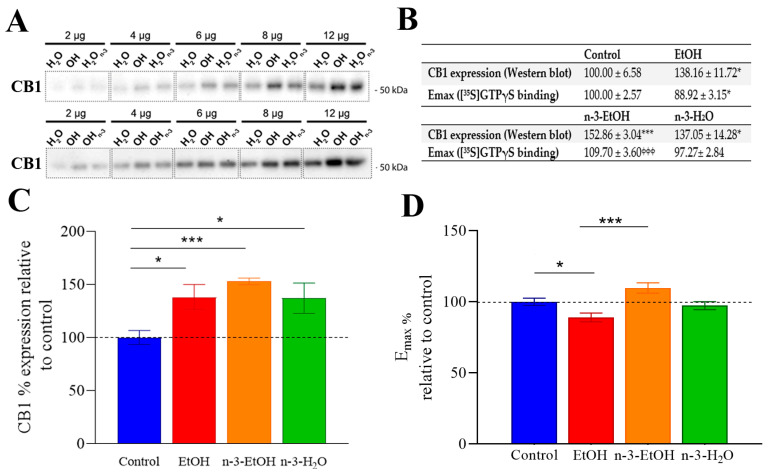
CB1 receptor expression and coupling to Gα_i/o_ in hippocampal synaptosomes from control, EtOH, n-3-EtOH and n-3-H_2_O mice. (**A**) Representative Western blot with increasing amounts of synaptosomal protein (2, 4, 6, 8, and 12 µg per lane). Protein loading was verified using Coomassie Brilliant Blue gel staining. The molecular weight of the immunoreactive band was determined using standard markers (indicated in the figure), and protein migration corresponded to the expected molecular mass (CB1, 52.8 kDa). H_2_O, OH, H_2_On-3 and OHn-3 correspond to control, EtOH, n-3-H_2_O and n-3-EtOH groups, respectively. (**B**) Analysis of CB1 relative expression and coupling to Gα_i/o_. CB1 expression values represent the means ± SEs of slopes (normalized to the control) obtained by linear regression analysis (see Supplementary Figure S2), and the E_max_ values represent the means ± SEs obtained from complete concentration–response curves (see Supplementary Figure S1). Both expression and Emax values were obtained from independent experiments using synaptosomal membranes prepared from three fractionation procedures, including hippocampal pools from at least six adult mice. (**C**) Histogram of relative CB1 expression (EtOH vs. control *p* = 0.0245; n-3-EtOH vs. control *p* = 0.0006; n-3-H_2_O vs. control *p* = 0.0476). (**D**) Histogram of Emax of CP 55,940-stimulated [^35^S]GTPγS binding (EtOH vs. control *p* = 0.0445; n-3-EtOH vs. EtOH *p* = 0.0006). The statistical significance was determined using the extra-sum-of-squares F test (F) method: * *p* < 0.05 and *** *p* < 0.001 compared to control; ^ϕϕϕ^ *p* < 0.001 compared to EtOH.

Densitometric analysis of immunoreactivity in synaptosomal membrane immunoblots revealed that CB1 receptor expression was significantly increased in the EtOH, n-3-H_2_O, and n-3-EtOH groups compared to controls (Figure 2A–C; Supplementary Figure S1).

To determine whether differences in CB1 receptor expression correlated with differences in receptor coupling to their cognate Gαi/o proteins, [^35^S]GTPγS binding assays using the cannabinoid agonist CP 55,940 were conducted. A lower maximal response (Emax) was detected in the EtOH group compared to controls (Figure 2B,D; Supplementary Figure S1). Consistent with our previous report [28], omega-3 supplementation did not directly affect Emax but reversed the EtOH-induced reduction, raising it above control levels (Figure 2B,D; Supplementary Figure S1). Thus, the upregulation of CB1 receptor expression in EtOH hippocampal synaptosomes contrasts with its reduced [^35^S]GTPγS binding capacity, further indicating functional impairment of CB1 receptors due to intermittent EtOH exposure during adolescence.

In conclusion, omega-3 supplementation during the withdrawal period improved CB1 receptor functionality, which may be partially explained by the observed increase in CB1 receptor expression in hippocampal synaptosomes.

For a more detailed understanding of the potential mechanisms underlying the changes in CB1–Gαi/o protein coupling, the expression levels of cannabinoid receptor-interacting protein 1a (Crip1a) and Gαi/o proteins were assessed via immunoblot analysis. Remarkably, Crip1a—a protein known to interact with CB1 receptors to modulate their functional state—showed a significant increase in expression in both the EtOH and n-3-H_2_O groups compared to controls. Interestingly, this increase was even more pronounced in the n-3-EtOH group (Table 1; Supplementary Figure S3).

Regarding the expression of Gαi/o protein subtypes, which act as canonical CB1 receptor signal transducers, the levels of Gαi_1_, Gαi_2_, and Gαi_3_ remained unchanged across groups, while Gαo was decreased only in the n-3-H_2_O group (Table 1; Supplementary Figure S3), consistent with our previous data [28]. Thus, CB1–Gαi/o protein coupling may be influenced more by changes in Crip1a expression than by alterations in Gαi/o protein levels.

### 2.3. MPP Excitatory Synaptic Transmission and Plasticity

**Figure 3:**
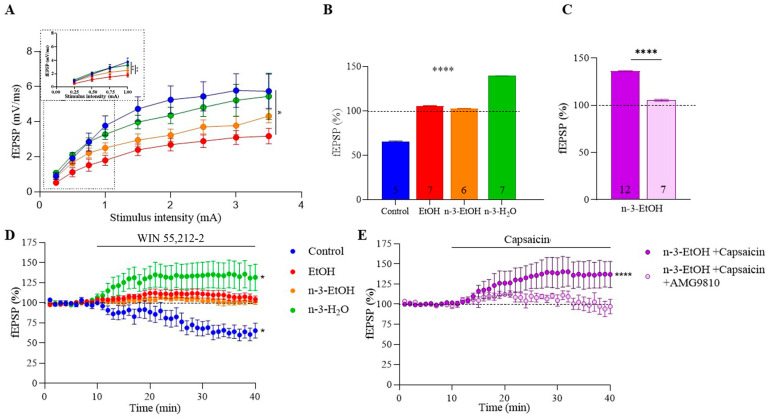
Omega-3 supplementation ameliorates the EtOH impact on the excitatory MPP synaptic transmission. (**A**) Input–output curves showing mean fEPSP areas (mV/ms) plotted against stimulation intensities in hippocampi of control (n = 13), EtOH (n = 17), n-3-EtOH (n = 20), and n-3-H_2_O (n = 21) mice (EtOH vs. control *p* = 0.0284). (**B**) Summary bar graphs of the WIN 55-212-2 effect (5 µM) on fEPSPs in control, EtOH, n-3-EtOH and n-3-H_2_O mice (EtOH vs. control *p* < 0.0001; n-3-EtOH vs. control *p* < 0.0001; n-3-H_2_O vs. control *p* < 0.0001; n-3-H_2_O vs. EtOH *p* < 0.0001; n-3-H_2_O vs. n-3-EtOH *p* < 0.0001). (**C**) Summary bar graphs for n-3-EtOH + capsaicin (1 µM) and n-3-EtOH + capsaicin (1 µM) + AMG9810 (3 µM) (n-3-EtOH+capsaicin+AMG9810 vs. n-3-EtOH+capsaicin *p* < 0.0001). (**D**) Time-course plot of the WIN 55,212-2 effect on fEPSPs in control (response vs. baseline *p* = 0.0373), EtOH, n-3-EtOH and n-3-H_2_O mice (response vs. baseline *p* = 0.0361). (**E**) Time-course plot of the capsaicin effect (1 µM) and AMG9810 (3 µM) on fEPSPs in n-3-EtOH (n-3-EtOH+capsaicin response vs. baseline *p* < 0.0001). Numbers in the bars are individual experiments. All data are presented as mean ± SEM and analyzed using two-way ANOVA and Tukey’s multiple comparison test (**A**,**B**), unpaired *t*-test (**C**) or paired *t*-test (**D**,**E**). Significant differences: * *p* < 0.05; ** *p* < 0.01,**** *p* < 0.0001.

To examine whether the loss of CB1-dependent synaptic transmission and LTD following adolescent binge drinking [3] could be reversed by omega-3 supplementation, the field excitatory postsynaptic potential (fEPSP) area in the MPP was analyzed. First, the slope of the input–output relationship (fEPSP amplitude response relative to stimulus intensity) was significantly decreased in EtOH mice (Figure 3A), supporting our previous findings [3]. Notably, the slope increased in EtOH mice on an omega-3 diet, suggesting that omega-3 mitigates the impairment in basal synaptic transmission caused by EtOH consumption (Figure 3A). Furthermore, the decrease in MPP excitatory synaptic transmission induced by the CB1 receptor agonist WIN 55,212-2 in control mice was absent in EtOH mice (Figure 3B,D; Supplementary Table S2), consistent with the loss of CB1 receptor functional coupling described above. Surprisingly, although omega-3 supplementation restored CB1 receptor coupling to Gαi/o proteins following EtOH exposure, it failed to reinstate the CB1-dependent reduction in MPP synaptic transmission (Figure 3B,D; Supplementary Table S2). Paradoxically, WIN 55,212-2 significantly increased MPP synaptic transmission in the n-3-H_2_O group [28].

**Figure 4:**
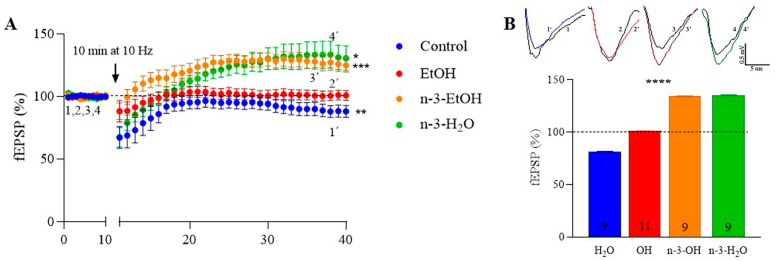
Omega-3-enriched diet potentiates MPP synaptic plasticity impaired by EtOH consumption during adolescence. (**A**) Effect of low-frequency stimulation (LFS; 10 min, 10 Hz) on fEPSPs in control (81.25 ± 0.46 **; response vs. baseline *p* = 0.0082), EtOH (101.00 ± 0.15), n-3-EtOH (134.20 ± 0.50 ***; response vs. baseline *p* = 0.0004) and n-3-H_2_O mice (135.40 ± 0.46 *; response vs. baseline *p* = 0.0181). Data are presented as mean ± SEM and analyzed using paired *t*-tests or the Wilcoxon test. Significance levels: * *p* < 0.05, ** *p* < 0.01, *** *p* < 0.001 compared to baseline. (**B**) Top: Superimposed traces of averaged fEPSPs from the last 10 min illustrating the LFS effect. Bottom: Summary bar graph showing MPP-LTD and MPP-LTP in control, EtOH, n-3-EtOH and n-3-H_2_O groups (EtOH vs. control *p* < 0.0001; n-3-EtOH vs. control *p* < 0.0001; n-3-H_2_O vs. control *p* < 0.0001; n-3-H_2_O vs. EtOH *p* < 0.0001; n-3-H_2_O vs. n-3-EtOH *p* < 0.0001). Numbers in the bars represent individual experiments. Data are expressed as mean ± SEM and analyzed using two-way ANOVA followed by Tukey’s multiple comparison test. Significance level: **** *p* < 0.0001.

Consistent with our previous studies [14,29], low-frequency stimulation (LFS; 10 min, 10 Hz) induced CB1 receptor- and 2-AG-dependent LTD at MPP synapses in control mice, but not in EtOH mice (Figure 4A,B) [3]. This loss of LTD could not be explained by changes in the expression of key enzymes involved in 2-AG synthesis—phospholipase Cβ1 (PLCβ1), DAGLα, and DAGLβ—whose levels in hippocampal synaptosomes remained unchanged compared to controls (Table 2; Supplementary Figure S4A–D). However, MAGL expression was increased in the EtOH group (Table 2; Supplementary Figure S4A,E), which may plausibly account for the LTD loss at MPP synapses and the reduced CB1 receptor coupling to Gαi/o proteins. Interestingly, LFS elicited LTP at MPP synapses in both omega-3 supplemented groups, a response markedly different from that seen in control and EtOH mice (Figure 4A,B).

**Figure 5:**
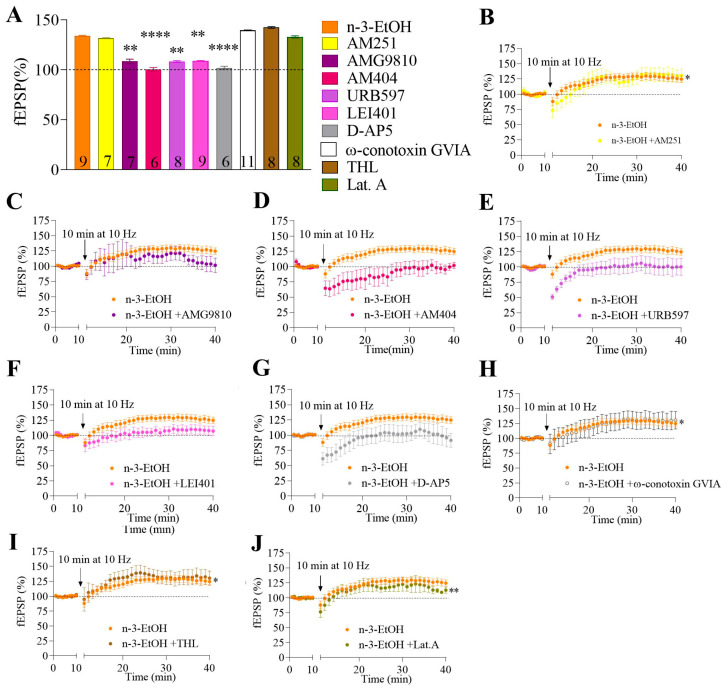
The MPP-LTP in n-3-EtOH mice is mediated by TRPV1, NMDA, and AEA. (**A**) The summary bar graph illustrates the effects of various pharmacological compounds (see the table in Section 4.7) on MPP-LTP: n-3-EtOH, n-3-EtOH + AM251 (4 µM), n-3-EtOH + AMG9810 (3 µM) (n-3-EtOH + AMG9810 vs. n-3-EtOH *p* = 0.0014), n-3-EtOH + AM404 (30 µM) (n-3-EtOH + AM404 vs. n-3-EtOH *p* < 0.0001), n-3-EtOH + URB597 (2 µM) (n-3-EtOH+URB597 vs. n-3-EtOH *p* = 0.0036), n-3-EtOH + LEI401 (10 µM) (n-3-EtOH+LEI401 vs. n-3-EtOH *p* = 0.0078), n-3-EtOH + D-AP5 (50 µM) (n-3-EtOH+D-AP5 vs. n-3-EtOH *p* < 0.0001), n-3-EtOH + ω-conotoxin GVIA (1 µM), n-3-EtOH + THL (10 µM), and n-3-EtOH + latrunculin A (500 µM). Numbers in the bars indicate individual experiments. Data are presented as mean ± SEM and analyzed using Dunn’s test. Significant differences versus n-3-EtOH LTP: ** *p* < 0.01, **** *p* < 0.0001. Time-course plots in n-3-EtOH mice in the absence or presence of (**B**) AM251 (response vs. baseline *p* = 0.0263), (**C**) AMG9810, (**D**) AM404, (**E**) URB597, (**F**) LEI401, (**G**) D-AP5, (**H**) ω-conotoxin GVIA (response vs. baseline *p* = 0.0425), (**I**) THL (response vs. baseline *p* = 0.0189), and (**J**) latrunculin A (response vs. baseline *p* = 0.0078). Data are presented as mean ± SEM and analyzed by paired *t*-test or Wilcoxon test, depending on the data distribution. Significant differences vs. baseline: * *p* < 0.05; ** *p* < 0.01.

We previously demonstrated that MPP-LTP in n-3-H_2_O mice is a presynaptic mechanism involving the CB1 receptor, 2-AG, and N-type Ca^2^⁺ channels [28]. Surprisingly, we found that the molecular players involved in MPP-LTP differed in n-3-EtOH mice compared to n-3-H_2_O mice. Consistent with the synaptic transmission data, MPP-LTP in n-3-EtOH mice was neither CB1 receptor nor 2-AG-dependent, as evidenced by the lack of effect of the CB1 receptor antagonist AM251 (Figure 5A,B; Supplementary Table S3) and the DAGL inhibitor THL (Figure 5A,I; Supplementary Table S3), respectively.

In n-3-EtOH mice, a marked reduction in DAGLα expression was observed in synaptosomes, along with a slight increase in PLCβ1 and DAGLβ expression (Table 2; Supplementary Figure S4A,B,D). However, this modest increase is unlikely to compensate for the sharp decline in DAGLα levels (Table 2; Supplementary Figure S4A,C). Furthermore, an increase in MAGL expression was detected (Table 2; Supplementary Figure S4A,E), suggesting reduced 2-AG production via the PLCβ1-DAGL pathway and explaining the absence of LTP dependence on 2-AG. Likewise, in n-3-EtOH mice, MPP-LTP was not affected by ω-conotoxin GVIA (an N-type Ca^2^⁺ channel blocker; Figure 5A,H; Supplementary Table S3) or by latrunculin A (an actin polymerization inhibitor; Figure 5A,I; Supplementary Table S3), indicating that presynaptic mechanisms were not involved, in contrast to n-3-H_2_O mice.

Finally, MPP-LTP was abolished by AMG9810 (a TRPV1 antagonist; Figure 5A,C; Supplementary Table S3), AM404 (an AEA transport inhibitor; Figure 5A,D; Supplementary Table S3), URB597 (a FAAH inhibitor; Figure 5A,E; Supplementary Table S3), LEI401 (a NAPE-PLD inhibitor; Figure 5A,F; Supplementary Table S3), and D-AP5 (an NMDA receptor antagonist; Figure 5A,G; Supplementary Table S3). These findings indicate the involvement of TRPV1, AEA, and NMDA receptors in the MPP-LTP observed in omega-3-supplemented mice following adolescent binge drinking. Consistently, the TRPV1 agonist capsaicin significantly increased MPP synaptic transmission in n-3-EtOH mice, an effect that was reversed by AMG9810 (Figure 5C,E; Supplementary Table S3). In regard to the role of AEA in synaptic plasticity, NAPE-PLD and FAAH expression levels in hippocampal synaptosomes were comparable across the experimental groups (Table 2; Supplementary Figure S4F,G).

### 2.4. Learning and Memory in the Barnes Maze Test

**Figure 6:**
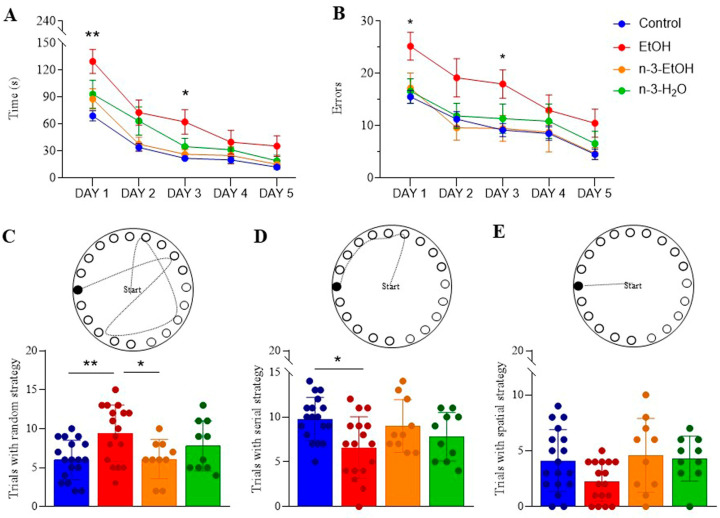
Barnes maze performance of control (n = 18), EtOH (n = 17), n-3-EtOH (n = 10) and n-3-H_2_O (n = 10) mice: (**A**) Time required (day 1: EtOH vs. control *p* = 0.0025; day 3: EtOH vs. control *p* = 0.0414) and (**B**) number of errors (day 1: EtOH vs. control *p* = 0.0217; day 3: EtOH vs. control *p* = 0.0300) to locate the escape box over five days of testing. Data are presented as mean ± SEM and analyzed using two-way ANOVA followed by Tukey’s multiple comparison test. Significance levels: * *p* < 0.05, ** *p* < 0.01 compared to control. (**C**) Random (EtOH vs. control *p* = 0.0046; n-3-EtOH vs. EtOH *p* = 0.0259), (**D**) serial (EtOH vs. control *p* = 0.0141), and (**E**) spatial strategies used by each experimental group. Data are expressed as mean ± SEM and analyzed using two-way ANOVA followed by Tukey’s multiple comparison test. Significance levels: * *p* < 0.05, ** *p* < 0.01.

The Barnes maze test was used to evaluate learning and memory in mice. Parameters measured included the time taken to locate the escape box, the number of errors made over five days of testing, and the type of strategy used. EtOH-exposed mice took significantly longer to find the escape box on the first day compared to controls (Figure 6A; Supplementary Table S4). They also made significantly more errors during the first three days (Figure 6B; Supplementary Table S5) and relied less on a serial strategy, instead using a random strategy more frequently than controls (Figure 6C,D). Omega-3 supplementation partially alleviated these deficits in EtOH-exposed mice by reducing the number of errors on the second day (Figure 6B; Supplementary Table S5) and decreasing the use of the random strategy (Figure 6C). However, no significant differences were observed in the use of the spatial strategy (Figure 6E). Moreover, omega-3 supplementation did not significantly affect the overall time to locate the escape box, the total number of errors, or strategy use compared to controls.

In summary, omega-3 intake mitigates some of the adverse effects of adolescent binge drinking on Barnes maze performance.

## 3. Discussion

Clarifying the role of the ECS in EtOH-induced neural signaling and synaptic disruptions in the brain may help identify novel pharmacological targets to mitigate the long-term effects of adolescent binge drinking and support the development of treatments for AUD [4,7,30,31,32,33,34]. Our findings demonstrate that omega-3 intake during adulthood modifies ECS function in the hippocampus following adolescent binge drinking. In particular, omega-3 increases CB1 receptor density at both excitatory and inhibitory terminals in the DG and counteracts the impaired CB1 receptor functionality caused by excessive EtOH consumption during adolescence. It also enhances the expression, in synaptic membranes (synaptosomes), of several enzymes involved in the metabolism of 2-AG, including PLCβ1, DAGLβ, and MAGL, as well as the CB1 receptor-associated protein Crip1a.

**Figure 7:**
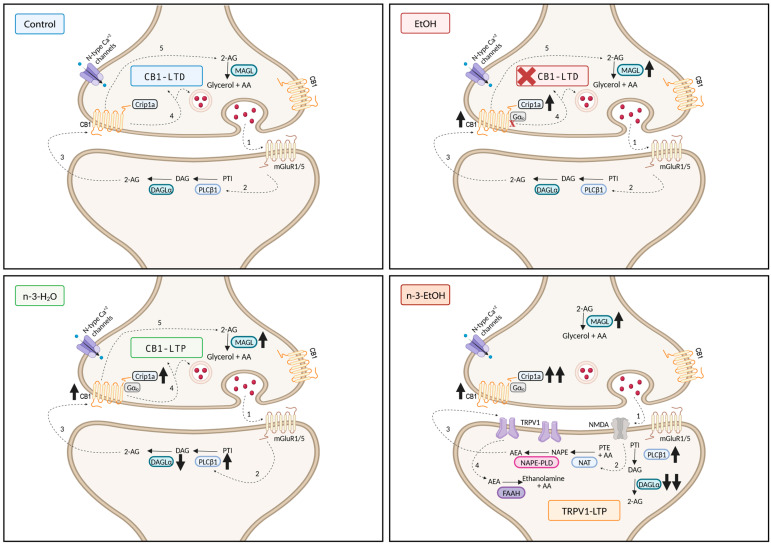
Summary of the main findings in the context of our previous reports [3,28,29]: Under physiological conditions (Control), LFS (10 Hz, 10 min) induces CB1-dependent LTD at excitatory MPP synapses in the DG. This form of synaptic plasticity requires the activation of group I mGluRs (1), which triggers the production of 2-AG by PLCβ1 and DAGLα (2), which then acts retrogradely on presynaptic CB1 receptors (3), leading to LTD (4). 2-AG is subsequently degraded by MAGL (5). Binge drinking during adolescence (EtOH) leads, after a period of abstinence, to an increase in CB1 receptors (↑), Crip1a (↑) and MAGL (↑) in synaptosomes, resulting in the abolishment of CB1-LTD and impaired cognitive performance in the Barnes maze test. Omega-3 supplementation during abstinence (n-3-EtOH) also increases CB1 receptors (↑), Crip1a (↑↑), MAGL (↑), and PLCβ1 (↑), while drastically decreasing DAGLα (↓↓) in synaptosomes. These changes in the ECS are associated with glutamate-activated NMDA receptors (1), which trigger AEA production (2) and postsynaptic TRPV1 activation (3). This leads to TRPV1-dependent LTP at the MPP synapses, resulting in a recovery of Barnes maze performance to levels comparable to control mice. AEA is subsequently degraded by FAAH (4). Omega-3 supplementation (n-3-H_2_O) also increases CB1 receptors (↑), Crip1a (↑), MAGL (↑), and PLCβ1 (↑), while decreasing DAGLα (↓) in synaptosomes. These changes in ECS components correlate with LTP at the MPP synapses. However, in contrast to n-3-EtOH, this form of synaptic plasticity is dependent on group I mGluRs (1), 2-AG synthesis (2), and CB1 receptors (3), ultimately leading to LTP (4). Subsequently, 2-AG is degraded by MAGL (5). Created in BioRender. Administrator, S. (2025) https://BioRender.com/g04m048 (accessed on 24 January 2025).

In contrast, omega-3 intake following adolescent binge drinking drastically reduces the expression of DAGLα, the primary enzyme responsible for 2-AG synthesis. Notably, DAGLα knockout mice have been shown to exhibit anxiogenic-like responses [11]. Nevertheless, the observed molecular and functional changes are associated with (1) the recovery of synaptic transmission and plasticity at excitatory MPP synapses, and (2) the restoration of Barnes maze performance to levels comparable to those of control mice. The molecular players underlying the LTP elicited by LFS (10 Hz, 10 min) at MPP synapses in control, EtOH, n-3-EtOH, and n-3-H_2_O mice are summarized in Figure 7.

### 3.1. Omega-3 Supplementation During Abstinence Alters ECS and CB1 Receptor Signaling Molecules Following Adolescent Binge Drinking

We used the chronic intermittent Drinking-in-the-Dark (DID) paradigm as a model to study binge-like EtOH consumption. This paradigm reliably induces high levels of EtOH intake and results in relevant blood ethanol concentrations (BEC). However, the effects of chronic EtOH exposure can vary depending on the specific exposure protocols and may be brain region specific [7].

Despite growing molecular evidence, there is still a lack of studies investigating at the synaptic level the functional consequences of changes in endocannabinoids caused by EtOH intake. Our findings demonstrate an increase in CB1 receptor expression following adolescent binge drinking, as well as after nutritional supplementation with an omega-3–enriched diet. Notably, CB1 receptor expression levels were comparable in mice that received omega-3 supplementation following EtOH exposure. After the withdrawal period, we observed an upregulation of CB1 expression, which, at the subcellular level, was associated with a modest increase in CB1 receptor density at excitatory terminals in the DG of n-3-EtOH mice compared to EtOH-only mice. Furthermore, there was a marked increase in CB1 receptor density at inhibitory terminals in both the EtOH and n-3-EtOH groups.

This increase in CB1 receptor expression is consistent with previous studies reporting CB1 upregulation following EtOH withdrawal [7,35]. However, in our recent study, CB1 optical density in the CA1 and DG regions of EtOH and n-3-EtOH mice did not differ significantly from controls [26]. These discrepancies may be attributed to differences in the resolution of the techniques used: electron microscopy and biochemical characterization of synaptosomal fractions allow for specific analysis of synaptic compartments, whereas immunohistochemistry detects CB1 receptor distribution at the cellular level.

Given that CB1 receptor density is approximately 10–20 times higher in inhibitory than in excitatory terminals in the hippocampus [36], the net increase in CB1 receptors observed in hippocampal synaptosomes in our study likely reflects the elevated CB1 density in inhibitory terminals. Thus, CB1 receptor enrichment in inhibitory synaptic membranes may contribute significantly to the overall receptor signal. Further studies are needed to elucidate the functional role of CB1 receptor enrichment in inhibitory and excitatory terminals following adolescent binge drinking and omega-3 supplementation.

Furthermore, CB1 receptor functionality was compromised, as evidenced by a decrease in the Emax of agonist-stimulated [^35^S]GTPγS binding in EtOH-exposed mice. Interestingly, this reduction in CB1 receptor activity occurred despite an upregulation of CB1 receptor expression in hippocampal synaptosomes, suggesting that excessive EtOH exposure during adolescence induces functional impairment of CB1 receptors. The observed reduction in CB1 receptor coupling to Gαi/o proteins caused by EtOH intake is consistent with previous studies [37,38,39]. However, these findings differ from our previous observations, which reported a decrease in CB1 receptor levels in hippocampal membrane preparations (P2 fraction) without significant changes in agonist-stimulated [^35^S]GTPγS binding efficacy [5]. These discrepancies highlight the importance of analyzing specific subcellular fractions, as they may reflect distinct regulatory mechanisms affecting CB1 receptor function depending on receptor localization.

One of the most challenging aspects to interpret is the apparent discrepancy between increased CB1 receptor expression and reduced functional coupling in the EtOH group, strongly suggesting the involvement of desensitization mechanisms. Importantly, expression levels of Gαo, Gαi1, Gαi2, and Gαi3 proteins were not significantly altered by EtOH exposure in our experiments, further suggesting that changes in G protein expression do not account for the observed functional impairment.

Since Crip1a interacts with CB1 receptors to reduce their signaling via Gαi/o proteins [40], the elevated Crip1a levels observed in the EtOH group could potentially counteract and partially explain the lack of increased CB1 signaling despite elevated receptor expression. In addition, with omega-3 supplementation, CB1 receptor functionality was restored, as indicated by a slight enhancement in the Emax value in n-3-EtOH mice compared to both control and EtOH groups in [^35^S]GTPγS binding assays. This restoration occurred despite the absence of significant differences in CB1 receptor or G protein expression levels relative to the EtOH group and in the presence of a slight further increase in Crip1a levels. These findings suggest that, beyond receptor expression and canonical signaling partners, additional yet unidentified mechanisms may contribute to the recovery of CB1 functionality following omega-3 supplementation.

Studies support the ability of lipids to modulate key aspects of G-protein coupled receptors, such as ligand-binding properties and the activation of downstream transducer proteins. These modulations can occur through specific interactions with the receptors or via alterations to the physicochemical properties of the lipid bilayer [41,42]. Thus, it is possible that CB1 dysfunction in the EtOH group—and its restoration by omega-3 supplementation—may be linked to changes in DHA levels within synaptic membranes. Indeed, EtOH consumption has been associated with a decrease in membrane DHA levels, whereas omega-3 supplementation restores them [43], which may help explain the observed functional recovery of CB1 receptors.

Our findings also indicate that adolescent binge drinking does not affect DAGLα expression in hippocampal synaptosomes. As previously discussed, 2-AG levels in n-3-EtOH mice may be altered, and together with elevated Crip1a levels, this could influence synaptic plasticity. Notably, MPP-LTP in n-3-EtOH mice was not mediated by 2-AG or CB1 receptors, unlike in control [29] and omega-3 supplemented mice [28]. This conclusion is supported by the observation that DAGLα inhibitors and CB1 antagonists failed to suppress MPP-LTP in these mice. Instead, MPP-LTP in n-3-EtOH mice was abolished by the TRPV1 antagonist AMG9810, and excitatory synaptic transmission was enhanced by TRPV1 activation. TRPV1 facilitates excitatory synaptic transmission and plays a role in hippocampal LTP [14,44,45,46]. In addition, omega-3 PUFAs and bioactive N-acylethanolamines, which are influenced by omega-3 intake, enhance TRPV1 expression and function [47]. Interestingly, mice exposed to environmental enrichment exhibited a similar shift from MPP-LTD to LTP, which also depended on TRPV1 activation and AEA, but not on CB1 receptors, group I mGluRs, or 2-AG [14]. These findings suggest that nutritional and environmental factors may recruit distinct molecular pathways to drive the switch to MPP-LTP.

EtOH intake has been shown to enhance TRPV1 and NMDA receptor activity [48,49] while simultaneously impairing NMDA-dependent synaptic plasticity—a deficit that can be reversed by CB1 receptor antagonism [50]. Binge drinking in adolescent rats has been shown to suppress LTD in hippocampal slices, resulting in learning impairments and increased NMDA receptor signaling through the GluN2B subunit [51]. NMDA receptor activation is essential for MPP-LTP, with particular dependence on the GluN2A and GluN2B subunits [52]. Both EtOH consumption and omega-3 deficiency disrupt long-term synaptic plasticity by altering NMDA receptor function, specifically through changes in GluN2A and GluN2B subunit expression [48,49,51]. However, the NMDA-dependent plasticity impairment caused by EtOH is notably reversed by omega-3 supplementation [21]. This effect may be attributed to DHA, which enhances NMDA receptor function by increasing channel open probability [53]. Indeed, the form of LTP enhanced by omega-3 fatty acids in EtOH-exposed male rodents, as observed in this and a previous study [21], depends on NMDA receptor activation. NMDA receptor function is closely tied to the cellular redox state, which is regulated by intracellular glutathione levels. Previous findings have shown that prenatal EtOH exposure reduces glutathione concentrations in the adult brain—a deficit that can be reversed through omega-3 supplementation [54]. Therefore, the MPP-LTP observed in our study may result, at least in part, from an omega-3–mediated increase in intracellular glutathione.

We did not observe changes in the expression of NAPE-PLD and FAAH. However, inhibitors of NAPE-PLD, FAAH, and AEA reuptake effectively abolished MPP-LTP, indicating that AEA mediates this form of synaptic plasticity in n-3-EtOH mice. There is strong interplay between TRPV1 and NMDA receptors. For instance, capsaicin activates NMDA-dependent ERK signaling, a pathway that is also enhanced by omega-3 supplementation [55]. Moreover, long-term NMDA-mediated synaptic plasticity impaired by EtOH can be restored by increasing AEA levels, which in turn activate TRPV1 [49]. NMDA receptors facilitate the calcium influx required for AEA production, and the lack of effect of latrunculin A on MPP-LTP in n-3-EtOH mice supports the interaction between NMDA and TRPV1 as a postsynaptic mechanism. This is corroborated by the abundant localization of TRPV1 in postsynaptic granule cell dendritic spines, which receive input from asymmetric MPP synapses in the DG [56].

### 3.2. Omega-3 Supplementation During Abstinence Mitigates Long-Lasting Cognitive Deficits Caused by Adolescent Binge Drinking

Our study revealed that EtOH-exposed mice required more time and made more errors locating the escape box during the Barnes maze test, primarily using a random search strategy. Omega-3 supplementation alleviated these deficits in EtOH mice by reducing errors in locating the escape box and decreasing their use of the random strategy. These results align with previous studies showing that n-3 supplementation improves learning and memory in both physiological and pathological conditions, particularly during development [57]. In addition, recent findings indicate that n-3 ameliorates learning and memory impairment caused by EtOH intake [43]. 

Omega-3 supplementation has also been shown to reverse synaptic plasticity impairments caused by prenatal EtOH exposure [21]. The effects of omega-3 supplementation on the ECS and synaptic plasticity may explain the recovery of deficits induced by adolescent binge drinking on Barnes maze performance, along with their anti-inflammatory properties and their role in promoting neurogenesis [43]. However, the mechanisms by which changes in the ECS lead to TRPV1-dependent LTP and ultimately improve cognitive function remain speculative. We suggest that the marked reduction of DAGLα and the significant increase in Crip1a might contribute to the shift toward TRPV1-dependent synaptic plasticity following omega-3 supplementation under EtOH conditions. Intracellular TRPV1 may regulate calcium release from the sarcoplasmic/endoplasmic reticulum, which is necessary for excitatory synaptic plasticity. The biosynthetic enzyme NAPE-PLD, responsible for producing AEA, is highly expressed in dentate granule cells and localized in postsynaptic dendrites and spines that receive excitatory synapses. The calcium-dependent catalytic activity of NAPE-PLD may generate AEA or other N-acylethanolamines, leading to postsynaptic TRPV1 activation. In addition, NMDA receptors could be triggering the Ca^2^⁺ increase needed for AEA production, which then acts at postsynaptic TRPV1. In fact, the lack of effect of latrunculin A on MPP-LTP in n-3-EtOH mice suggests a postsynaptic mechanism, reinforced by the abundant localization of TRPV1 at postsynaptic granule cell dendritic spines of asymmetric perforant path synapses in the dentate molecular layer [56]. Remarkably, mice lacking TRPV1 display learning deficits associated with reduced excitatory LTP [45].

### 3.3. Experimental Limitations

This study has some limitations that should be addressed in future research to better elucidate the effects of omega-3 on EtOH-induced damage to the hippocampal ECS and its subsequent impact on hippocampal functions. First, the absence of an analysis of 2-AG and AEA levels limits our understanding of how omega-3 modulates specific endocannabinoid pathways affected by EtOH exposure. Second, the lack of data on neuronal phospholipid membrane composition hinders the evaluation of potential structural changes induced by omega-3 supplementation, which may underlie the observed functional effects. Ongoing research in our laboratory includes advanced lipidomics to investigate phospholipid dynamics in neuronal membranes and comprehensive endocannabinoid quantification to provide a more detailed understanding of the system’s recovery. These approaches may offer critical insights into the molecular mechanisms by which omega-3 fatty acids exert their protective effects and help identify more precise therapeutic targets for mitigating alcohol-related brain damage.

Binge drinking by women is likely to impose a greater burden on healthcare systems and increase the social stigma associated with alcohol abuse by women, ultimately affecting society as a whole. We are currently examining sex and gender differences in alcohol’s effects, its consequences, and the potential of nutritional supplementation in the prevention and/or treatment of alcohol-related disorders in women, since men and women respond differently to alcohol, exhibiting distinctive effects on cognition [58].

## 4. Materials and Methods

### 4.1. Ethics Statement

The protocols for animal care and use were approved by the Committee of Ethics for Animal Welfare of the University of the Basque Country (M20-2020-113; date of approval: 29 September 2020). They also complied with the European Communities Council Directive of 22 September 2010 (2010/63/EU) and Spanish regulations (Real Decreto 53/2013, BOE 8 February 2013). The number of animals and the suffering were controlled and minimized.

### 4.2. Animal Treatment

**Figure 8:**
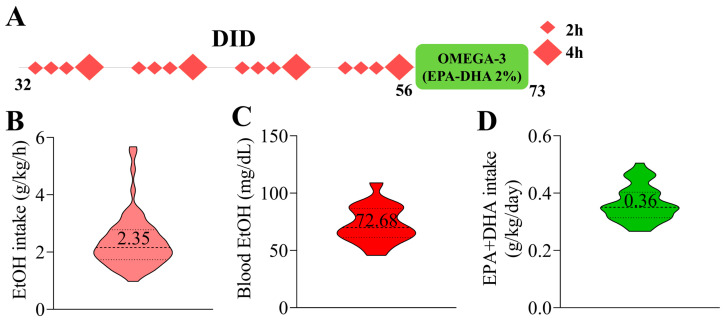
Schematic representation of the experimental timeline, total EtOH intake, BEC, and EPA+DHA intake. (**A**) Male C57BL/6J mice were subjected to a 4 week DID protocol during adolescence (PND 32–56). The mice were given free access to 20% (*v*/*v*) EtOH each week for 2 and 4 h. During withdrawal (PND 56–73), half of the mice were provided with an omega-3-enriched diet. (**B**) The total EtOH intake was 2.35 ± 0.11 g/kg/h (n = 18). (**C**) The BEC measured on the final day of EtOH exposure was 72.68 ± 3.78 mg/dL (n = 18). (**D**) The average daily intake of EPA+DHA was 0.36 ± 0.01 g/kg/day (n = 30).

Four-week-old C57BL/6J adolescent male mice (Janvier Labs, Le Genest-Saint-Isle, France) were randomly housed in pairs and assigned to either the H_2_O (control) or EtOH groups. The mice underwent a DID procedure for four weeks, spanning postnatal days (PND) 32 to 56 (Figure 8A). Briefly, on days 1–4 of each week, each mouse was individually presented with a 10 mL bottle containing either tap water or an EtOH solution (20% *v*/*v* EtOH, prepared from 96% EtOH; Boter S.L., Barcelona, Spain). Mice had unrestricted access to the bottle for 2 h on the first three days and for 4 h on the fourth day. They were provided with food and water ad libitum during the remaining three days of each week.

The effectiveness of the DID procedure was evaluated by measuring total EtOH intake (g/kg/h) (Figure 8B). Blood samples were collected from the lateral tail vein 30 min after the final exposure on the last day of each DID week. The BEC (mg/dL) was then measured using a commercial EtOH assay kit (Abcam ab65343, Cambridge, UK) (Figure 8C).

During the withdrawal period (PND 57–73), half of the mice were randomly selected and given a diet enriched with 2% EPA and DHA (1.2% EPA and 0.8% DHA—SAFE, Augy, France; Table 3), referred to as the n-3-EtOH and n-3-H_2_O groups. Twice a week, mice and food were weighed to assess EPA and DHA intake (mg/kg/day) (Figure 8D), and the food was replaced regularly to prevent fat oxidation. During the final days of the withdrawal period, separate groups of mice underwent either electrophysiological recordings or the Barnes maze test. Mice subjected to the behavioral test were sacrificed on PND 73, and their brains were subsequently processed for synaptosomal fractionation or electron microscopy analyses.

### 4.3. Pre-Embedding Immunogold and Immunoperoxidase for Electron Microscopy

On PND 73, three mice per group were euthanized. Mice were deeply anesthetized with 4% chloral hydrate (10 mL/kg body weight, administered intraperitoneally) and perfused through the left ventricle with 30 mL phosphate-buffered saline (PBS, 0.1 M, pH 7.4), followed by 80 mL of fixative solution (4% formaldehyde depolymerized from paraformaldehyde, 0.2% picric acid, and 0.1% glutaraldehyde) prepared in PBS at room temperature (RT). Brains were then extracted and post-fixed in the same fixative solution at 4 °C for one week before being transferred to a 1:10 diluted fixative solution until further processing.

The pre-embedding immunogold and immunoperoxidase methods were applied simultaneously and repeated three times on sections obtained from each mouse in the control (n = 3), EtOH (n = 3), n-3-H_2_O (n = 3), and n-3-EtOH (n = 3) groups. Briefly, 50 µm-thick coronal vibratome sections of the hippocampus were pre-incubated for 30 min at RT in 10% bovine serum albumin (BSA) and Tris-HCl buffered saline (TBS) (pH 7.4) containing 0.1% sodium azide and 0.02% saponin. Next, sections were incubated with primary antibodies targeting the CB1 receptor and GLAST (Supplementary Table S6) in 10% BSA/TBS with 0.1% sodium azide and 0.004% saponin. This incubation was carried out with gentle shaking for two days at 4 °C. After thorough washing, sections were incubated with secondary antibodies (Supplementary Table S6) and placed in an avidin–biotin complex solution (1:50, PK-7100, Vector Labs, Newark, CA, USA) for 1.5 h at RT. After overnight washing, sections were post-fixed in 1% glutaraldehyde in TBS for 10 min at RT.

Gold particles were then silver enhanced using an HQ Silver Enhancement Kit (Nanoprobes Inc., Yaphank, NY, USA) for 12 min in the dark. Following this, sections were treated with 0.05% 3,3′-diaminobenzidine tetrahydrochloride and 0.01% hydrogen peroxide in 0.1 M phosphate buffer (PB) for 3 min at RT. After multiple washes, stained sections were post-fixed in 1% osmium tetroxide in 0.1 M PB for 20 min, dehydrated in a graded series of alcohols (50–100%) followed by propylene oxide, and embedded in Epon resin 812. Finally, ultrathin sections (50 nm) were cut with a diamond knife (Diatome, Nidau, Switzerland), collected on nickel mesh grids, and stained with 2.5% lead citrate for 20 min. These sections were examined using an electron microscope (JEOL JEM 1400 Plus, Tokyo, Japan) and imaged with a digital camera (Morada, sCMOS, Olympus, Tokyo, Japan). A total of 24 hippocampi were analyzed, six from each group. Electron micrographs were randomly taken at 8000× magnification, covering a total analyzed area of approximately 1100 µm^2^ per mouse, with a comparable area examined in the control group. The numbers of excitatory and inhibitory terminals, as well as GLAST-stained astrocytes analyzed, were consistent across mice in the experimental groups (Supplementary Table S7). After image analysis, the percentage of CB1-immunopositive profiles (the number of CB1-positive profiles divided by the total number of identified profiles) and CB1 density (the number of gold particles divided by the perimeter of the profile) were calculated.

### 4.4. Synaptosomal Fractionation

After sacrifice by cervical dislocation at PND 73, brains were extracted, and the hippocampi were dissected and stored at −80 °C until use. Considering that the yield of fractionation is about 1–1.5% (1–1.5 mg synaptosomal protein per 100 mg fresh tissue), pooled hippocampal tissue from at least six mice (about 160 mg fresh tissue weight) was used per fractionation procedure.

Hippocampal tissue was thawed slowly on ice-cold 0.32 M sucrose, pH 7.4, containing 80 mM Na_2_HPO_4_ and 20 mM NaH_2_PO_4_ (sucrose PB), and then homogenized in 10 volumes of sucrose/PB using a motor-driven Potter Teflon glass homogenizer (motor speed 800 rpm; 10 up-and-down strokes; cooled in an ice-water mixture throughout). The homogenate was initially centrifuged at 1000× *g* for 10 min, and the resulting supernatant (S1) was further centrifuged at 15,000× *g* for 30 min. The resulting pellet (P2) was resuspended, centrifuged again at 15,000× *g* for 30 min, resuspended to a final volume of 16 mL, and transferred into a centrifugation tube layered with 8 mL of 1.2 M sucrose/PB at the bottom. This mixture was then centrifuged at 180,000× *g* for 30 min. The material retained at the gradient interface was collected and diluted with 0.32 M sucrose/PB to a final volume of 16 mL. This diluted sample was layered onto 8 mL of 0.8 M sucrose/PB and centrifuged at 180,000× *g* for another 30 min. The resulting pellet was resuspended and subjected to a final centrifugation at 40,000× *g* for 30 min. The synaptosomal pellets obtained were then frozen at −80 °C. Protein content was determined using the Bio-Rad dye reagent with a bovine globulin standard.

### 4.5. Western Blot Analysis

Synaptosomal membranes from the experimental groups were thawed and processed in parallel. Samples were heated for 5 min at 60 °C in urea-denaturing buffer (20 mM Tris-HCl, pH 8.0, 12% glycerol, 12% urea, 5% DTT, 2% SDS, 0.01% bromophenol blue). Denatured proteins were then separated by electrophoresis on 10% SDS–polyacrylamide (SDS–PAGE) gradient gels using the Mini Protean II gel apparatus (Bio-Rad, Hercules, CA, USA). Proteins were subsequently transferred to polyvinylidene fluoride (PVDF) membranes (Amersham Bioscience, Buckinghamshire, UK) for immunoblotting, using the Mini Trans-Blot transfer unit (Bio-Rad, Hercules, CA, USA) at 30 V overnight at 4 °C.

Following transfer, membranes were blocked for 1 h at RT in 5% non-fat dry milk in PBS containing 0.5% BSA and 0.2% Tween 20. Membranes were then incubated with the corresponding primary antibodies diluted in blocking buffer at appropriate concentrations (Supplementary Table S8). Next, blots were incubated with specific horseradish peroxidase-conjugated secondary antibodies diluted 1:10,000 in blocking buffer for 2 h at RT. Immunoreactive bands were revealed with the enhanced chemiluminescence (ECL) system according to the manufacturer’s instructions (Amersham Bioscience, Buckinghamshire, UK) and acquired using an ImageQuant 350 imager device (Cytiva, Marlborough, MA, USA). Densitometric analysis of digital immunoreactive signals was performed using ImageJ software (version 1.8.0_322; NIH, Bethesda, MD, USA).

Our approach provides an internal loading control by including increasing amounts of protein per condition. Linear regression analysis of the immunoreactive signal enables detection and correction of minor loading inconsistencies, which would appear as deviations from linearity. In addition, denatured samples intended for immunodetection were run on separate SDS–PAGE gels, stained with Coomassie Blue, and analyzed by densitometry to correct the optical density (OD) values of immunoreactive bands, thereby ensuring equal protein loading.

### 4.6. [^35^S]GTPγS Binding Assays

Hippocampal synaptosomes (each sample corresponding to a pool from 6–8 mice) were thawed and incubated (5 µg of total protein per tube) at 30 °C for 2 h in [^35^S]GTPγS incubation buffer (0.5 nM [^35^S]GTPγS, 1 mM EGTA, 3 mM MgCl_2_, 100 mM NaCl, 0.2 mM DTT, 50 μM GDP, 0.5% fatty acid-free BSA, and 50 mM Tris-HCl, pH 7.4). Basal binding was defined as the specific [^35^S]GTPγS binding in the absence of an agonist. The CB1 receptor agonist CP55,940 (1 nM–10 µM) was added to determine receptor-stimulated [^35^S]GTPγS binding, and nonspecific binding was estimated in the presence of 10 µM unlabeled GTPγS. Reactions were terminated by rapid vacuum filtration through Whatman GF/B glass fiber filters, and the bound radioactivity was measured by liquid scintillation spectrophotometry. The concentration-dependent increase in specific [^35^S]GTPγS binding induced by CP55,940 was expressed as a percentage of the basal, unstimulated value. Data were analyzed by nonlinear regression to determine the Emax and the concentration required to elicit the half-maximal effect (pEC_50_).

### 4.7. Slice Preparation and Extracellular Field Recording

Adult C57BL/6J mice (PND 67–71) were anesthetized via isoflurane inhalation and their brains were quickly extracted and placed in a chilled, sucrose-based solution (4 °C) containing 87 mM NaCl, 75 mM sucrose, 25 mM glucose, 7 mM MgCl_2_, 2.5 mM KCl, 0.5 mM CaCl_2_, and 1.25 mM NaH_2_PO_4_. Coronal brain slices (300 μm thick) were prepared using a vibratome (Leica VT1000s; Leica Biosystems, Nussloch, Germany), then recovered at 35 °C and continuously superfused (2 mL/min) in the recording chamber with artificial cerebrospinal fluid (ACSF: 130 mM NaCl, 11 mM glucose, 1.2 mM MgCl_2_, 2.5 mM KCl, 2.4 mM CaCl_2_, 1.2 mM NaH_2_PO_4_, and 23 mM NaHCO_3_) equilibrated with 95% O_2_/5% CO_2_. The superfusion medium included picrotoxin (100 μM) to block GABA-A receptors and additional drugs (Table 4) were added at their final concentrations.

For extracellular field recordings, the stimulating electrode was positioned in the MPP and the recording pipette was placed in the inner third of the DG. After recording a stable baseline, LFS (10 min at 10 Hz) was applied to induce eCB-mediated long-term plasticity of glutamatergic inputs. The area of the fEPSP was measured. The magnitude of long-term plasticity following LFS was calculated as the percentage change between the baseline (average excitatory responses over 10 min prior to LFS) and the final 10 min of stable responses, recorded 30 min after LFS ended. The slices analyzed (n) were obtained from at least three different mice.

### 4.8. Barnes Maze Test

To evaluate learning and memory, at least 10 animals per experimental group were tested in the Barnes maze. The setup consisted of a table with 20 evenly spaced holes placed in the center of a room with various visual cues. Each mouse was assigned an escape box located at a randomly designated hole, which remained consistent throughout the test. Over five consecutive days (PND 67–71) each mouse completed four trials per day to locate the escape box. Mice had a maximum of 240 s per trial to find the escape box, after which they were allowed to rest inside it for 60 s.

To locate the escape box, mice could use different strategies: random, serial, or spatial. Data collected included escape latency, the number of errors (non-target holes visited before finding the escape box), and the strategy used.

### 4.9. Statistical Analysis

Statistical analyses were performed using GraphPad Prism 8 (GraphPad Software; RRID: SCR_002798). All values are presented as mean ± SEM, except for Western blots and [^35^S]GTPγS binding assays, which are shown as mean ± SE. A significance threshold of *p* < 0.05 was set for all comparisons.

For electron microscopy and Barnes maze data, two-way ANOVA followed by Tukey’s multiple comparisons test was performed. For electrophysiological experiments, various statistical analyses were applied. Time-course data were analyzed using either paired t-tests or Wilcoxon signed-rank tests, depending on data distribution (assessed with the Shapiro–Wilk normality test). To compare the effects among the four experimental groups, two-way ANOVA followed by Tukey’s multiple comparisons test was used. Dunn’s test was applied to analyze the effects of different drugs on MPP-LTP within the n-3-EtOH group.

In Western blot assays, data were obtained from three independent biological replicates (n = 3) corresponding to synaptosomal preparations from three distinct pools of 6–8 mice each. Increasing amounts of total protein from each sample were resolved and processed in parallel for immunoblotting. Densitometric analysis of specific immunoreactive bands yielded one raw integrated OD value per protein load per sample. These values were normalized to the highest protein amount from control synaptosomal samples. For each preparation, technical replicates were progressively accumulated and averaged per protein load point until the regression fit reached a minimum threshold (R^2^ > 0.95). These cumulative load-point averages were then used to construct a single regression curve for each biological replicate. This approach ensured accurate determination of the linear detection range for each antibody and enabled relative quantification of protein expression between experimental groups by comparing the slopes of the resulting regression lines. Differences between slopes were evaluated using pairwise F-tests and *p*-values were adjusted using the Holm–Bonferroni method to correct for multiple comparisons.

In the [^35^S]GTPγS binding assays, data from independent biological replicates (n = 3 synaptosomal fractionations; 6–8 mice per fractionation) were used as individual data points to generate a CP55,940 concentration–response curve, fitted by nonlinear regression using the four-parameter Hill equation. EC_50_ values were logarithmically transformed for statistical analysis, as affinity constants derived from such assays typically follow a log-normal distribution. Statistical significance of differences between the means of parameter estimates was evaluated using F-tests, and the resulting *p*-values were adjusted using the Holm–Bonferroni method to correct for multiple comparisons.

## Figures and Tables

**Table 1:** Relative expression of CB1 receptor, Gα_i/o_ proteins and Crip1a in hippocampal synaptosomes from control, EtOH, n-3-EtOH and n-3-H_2_O groups determined by the slope comparison method.

	Control	EtOH	n-3-EtOH	n-3-H_2_O
CB1 receptor	100.00 ± 6.58	138.16 ± 11.72 *	152.86 ± 3.04 ***	137.05 ± 14.28 *
Crip1a	100.00 ± 9.44	142.91 ± 13.27 *	194.56 ± 19.08 ***	149.96 ± 12.53 ***
Gα_o_	100.00 ± 1.75	92.88 ± 2.48	84.53 ± 4.98	67.22 ± 8.86 *
Gα_i1_	100.00 ± 9.10	98.56 ± 6.81	91.00 ± 7.07	84.68 ± 5.59
Gα_i2_	100.00 ± 10.94	128.07 ± 15.63	91.78 ± 1.05	102.10 ± 6.52
Gα_i3_	100.00 ± 0.11	93.88 ± 5.60	97.63 ± 3.98	74.49 ± 10.65

Values represent the means ± SEs of slopes (normalized to control) obtained by simple regression analysis (Supplementary Figure S2) from independent experiments using synaptosomal membranes from three fractionation procedures and including hippocampal pools from at least six adult mice per fractionation procedure. The statistical significance between slopes was determined using the extra-sum-of-squares F test (F) method: * *p* < 0.05,*** *p* < 0.001 compared to control.

**Table 2:** Relative expression of the principal enzymes involved in 2-AG and AEA synthesis and degradation in hippocampal synaptosomes from control, EtOH, n-3-EtOH and n-3-H_2_O mice determined by the slope comparison method.

	Control	EtOH	n-3-EtOH	n-3-H_2_O
PLCβ1	100.00 ± 5.52	108.81 ± 12.243	138.29 ± 9.55 **	129.69 ± 7.92 **
DAGLα	100.00 ± 3.68	106.82 ± 3.43	20.42 ± 1.93 ***^ϕϕ^	47.88 ± 10.29 ***^ϕϕϕϯϯ^
DAGLβ	100.00 ± 12.11	88.45 ± 16.12	147.39 ± 15.31	135.91 ± 16.12
MAGL	100.00 ± 8.78	150.46 ± 14.23 *	150.73± 15.47 *	132.74 ± 7.41 *
NAPE-PLD	100.00 ± 6.60	118.94 ± 11.82	87.31 ± 11.61	108.81 ± 18.54
FAAH	100.00 ± 9.50	113.29 ± 11.01	106.84 ± 13.41	99.02 ± 4.09

Values represent the means ± SEs of slopes (normalized to control) obtained by simple regression analysis (Supplementary Figure S2) from independent experiments using synaptosomal membranes from three fractionation procedures and including hippocampal pools from at least six adult mice per fractionation procedure. The statistical significance between slopes was determined using the extra-sum-of-squares F test (F) method: * *p* < 0.05, ** *p* < 0.01, *** *p* < 0.001 compared to control; ^ϕϕ^ *p* < 0.01, ^ϕϕϕ^ *p* < 0.001 compared to EtOH; ^ϯϯ^ *p* < 0.01 compared to n-3-EtOH.

**Table 3:** Comparison of the fatty acid composition between standard and omega-3 enriched diets.

	Standard Diet		N-3 Enriched Diet	
Fats (%)	4.0		5.9	
	mg/kg	% of total fats	mg/kg	% of total fats
SFA	6000	15.0	11,867	20.1
PUFA	21,000	52.5	31,129	52.8
Omega-3	1000	2.5	5437	9.2
Omega-6	20,000	50.0	25,679	43.5
Ratio omega-6/omega-3	-	20.0	-	4.7
EPA	0	0	1325	2.2
DHA	0	0	899	1.5

SFA = Saturated fatty acids; PUFA = Polyunsaturated fatty acids.

**Table 4:** Drugs used in electrophysiology.

Drug	Action	Concentration (μM)
AM251	CB1 antagonist	4
AMG9810	TRPV_1_ antagonist	3
AM404	AEA reuptake inhibitor	30
URB597	FAAH inhibitor	2
LEI401	NAPE-PLD inhibitor	10
D-AP5	NMDA antagonist	50
Lat.A	Actin assembly inhibitor	500
ω-conotoxin GVIA	N-type Ca^2+^ channels blockade	1
THL	DGL inhibitor	10
WIN-2	CB1 agonist	5
Capsaicin	TRPV_1_ agonist	1

## Data Availability

The raw data supporting the conclusions of this article will be made available by the authors on request.

## Supplementary Materials

The supporting information can be downloaded at: https://www.mdpi.com/article/10.3390/ijms26125507/s1.
