# Supplementary material for: Omega-3 Fatty Acids Mitigate Long-Lasting Disruption of the Endocannabinoid System in the Adult Mouse Hippocampus Following Adolescent Binge Drinking
Source: Int J Mol Sci. 2025 Jun 9;26(12):5507. doi: 10.3390/ijms26125507 (PMC12192632; doi:10.3390/ijms26125507)
Supplement: Supplementary file 1 [file ijms-26-05507-s001.zip › ijms-3592958-Supplementary.pdf]

International Journal of *Molecular Sciences*

MDPI

# Supplementary Information

# OMEGA-3 FATTY ACIDS MITIGATE LONG-LASTING DISRUPTION OF THE ENDOCANNABINOID SYSTEM IN THE ADULT MOUSE HIPPOCAMPUS FOLLOWING ADOLESCENT BINGE DRINKING

**Maitane Serrano [1,2,3], Miquel Saumell-Esnaola [4,5], Garazi Ocerin [1,2], Gontzal García del Caño [5,6], Edgar Soria-Gómez [1,2,3,7], Amaia Mimenza [1,2,3], Nagore Puente [1,2,3], Itziar Bonilla-Del Río [1,2,3], Almudena Ramos [1,2,3], Leire Reguero [1,2,3], Brian R. Christie [8,9,10], Inmaculada Gerrikagoitia [1,2,3] and Pedro Grandes [1,2,3]**

1 Department of Neurosciences, Faculty of Medicine and Nursing, University of the Basque Country UPV/EHU, Leioa, 48940, Spain
2 Achucarro Basque Center for Neuroscience, Science Park of the UPV/EHU, Leioa, 48940, Spain
3 Atención Primaria, Cronicidad y Promoción de la Salud, Red de Investigación en Atención Primaria de Adicciones (RIAPAd), RD21/0009/0006, Leioa, Spain
4 Department of Pharmacology, Faculty of Pharmacy, University of the Basque Country UPV/EHU, Vitoria-Gasteiz, Spain
5 Bioaraba, Neurofarmacología Celular y Molecular, Vitoria-Gasteiz, Spain
6 Department of Neurosciences, Faculty of Pharmacy, University of the Basque Country UPV/EHU, Vitoria-Gasteiz, Spain
7 Ikerbasque, Basque Foundation for Science
8 Division of Medical Sciences and Institute for Aging and Lifelong Health, University of Victoria, Victoria, BC, Canada
9 Island Medical Program and Department of Cellular and Physiological Sciences, University of British Columbia, Vancouver, BC, Canada
10 Department of Psychology, San Diego State University, San Diego, CA, USA

## Supplementary Figures

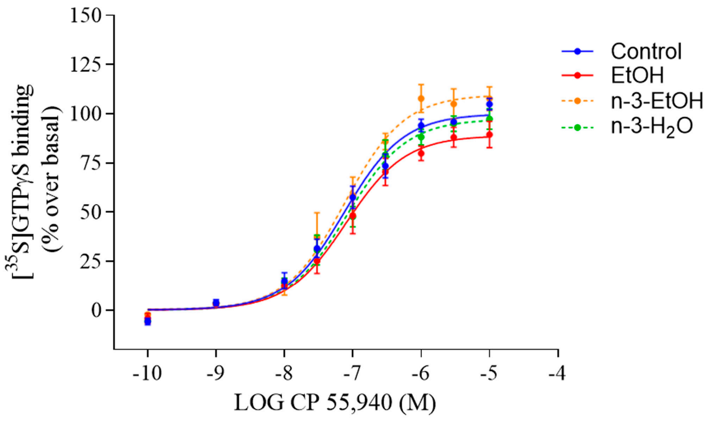


**Supplementary Figure S1. CB1 receptor coupling to G$\alpha_{i/o}$ proteins in hippocampal synaptosomes from control, EtOH, n-3-EtOH and n-3-H$_2$O groups.** Concentration–response curves for CP 55,940-stimulated [$^{35}$S]GTP$\gamma$S binding. Data represents the means ± SE from three independent

experiments, using synaptosomal membranes prepared through three fractionation procedures, with each fractionation including hippocampal samples pooled from at least six adult mice. Maximal response ($E_{max}$) and potency ($pEC_{50}$) values are expressed as % specific $[^{35}S]GTP\gamma S$ bound of basal. **$E_{max}$:** Control 100.00 ± 2.57; EtOH 88.92 ± 3.15*; n-3-EtOH 109.70$^{\phi\phi\phi}$ ± 3.60; n-3-$H_2O$ 97.27 ± 2.84. **$pEC_{50}$:** Control 7.12 ± 0.06; EtOH 7.10 ± 0.09; n-3-EtOH 7.11 ± 0.09; n-3-$H_2O$ 7.08 ± 0.08. The statistical significance between $E_{max}$ and $pEC_{50}$ parameters was determined using the extra-sum-of-squares F test (F) method. This method compares the goodness-of-fit of the experimental data for all groups combined (null hypothesis: $E_{max}$ or $pEC_{50}$ is the same across all datasets) against the goodness-of-fit for each group analyzed individually (alternative hypothesis: $E_{max}$ or $pEC_{50}$ differs across datasets).

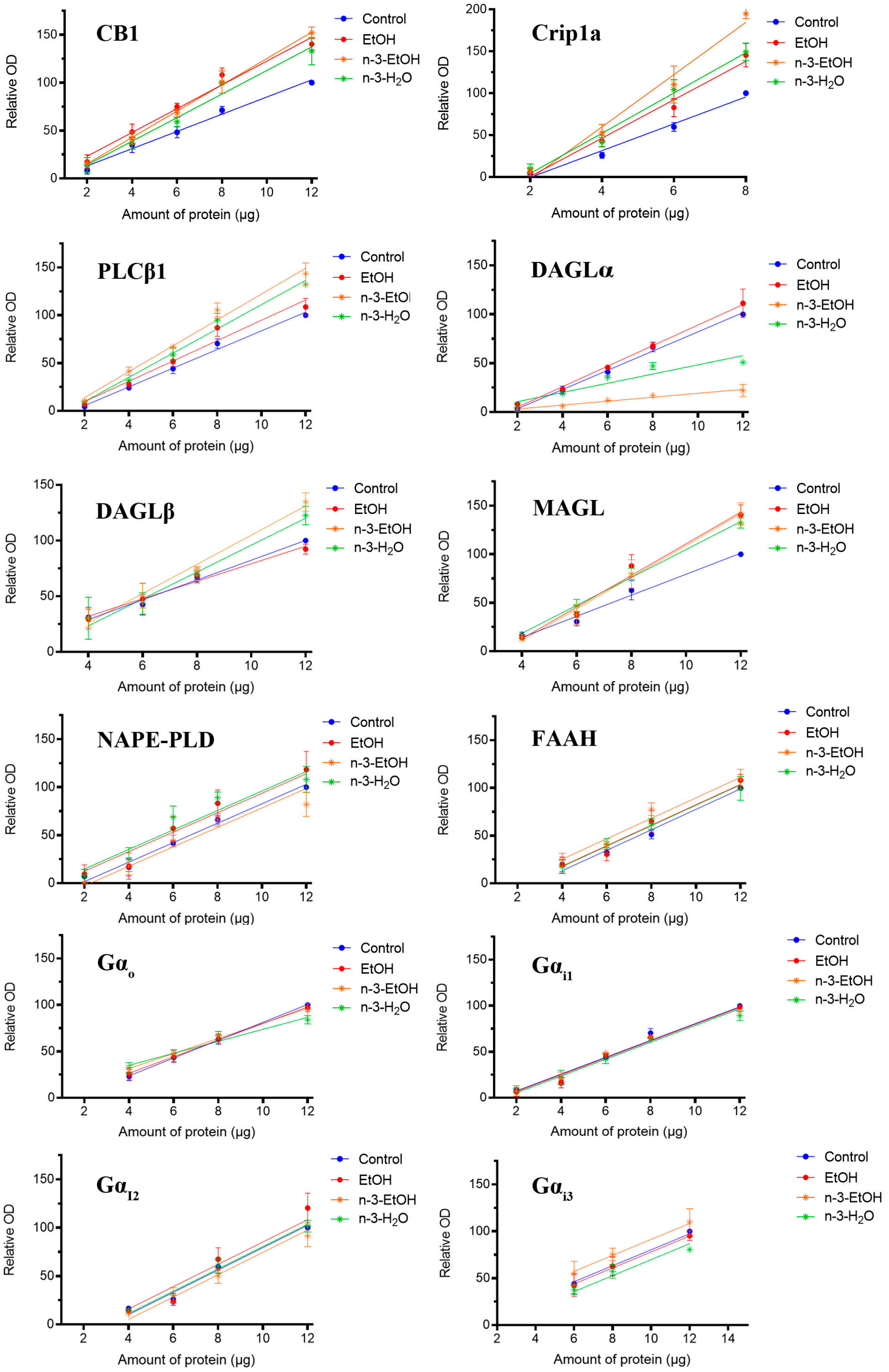

**Supplementary Figure S2. Regression analysis of immunoreactive signal values (integrated optical density) corresponding to increasing amounts of synaptosomal total protein**. Regression analysis provided the equation of a simple linear regression model. The resulting slopes of the equations were subsequently used to determine the relative protein expression. Regression analysis data are presented as mean ± SE employing synaptosomal membranes obtained from three fractionation procedures and including hippocampal pools from at least six adult mice per fractionation procedure. CB1 receptor: control 8,98 – 4,80; EtOH 12,41 - 1,58; n-3-EtOH 13,731 - 12,06; n-3-$H_2O$ 12,31 – 10,35; Crip1a: control 16,01 - 32,52; EtOH 22,88 - 45,06; n-3-EtOH 31,15 - 64,66; n-3-$H_2O$ 24,01 - 43,74 PLCβ1: control 9,76 - 13,94; EtOH 10,63 - 11,54 n-3-EtOH 13,51 - 12,94; n-3-$H_2O$ 12,67 - 15,56. DAGL$\alpha$: control 9,82 - 16,20; EtOH 10,49 - 15,94; n-3-EtOH 2,00 - 0,83; n-3-$H_2O$ 4,70 - 1,27; DAGLβ: control 8,88 - 5,41; EtOH 7,86 + 0,61; n-3-EtOH 13,10 - 26,07; n-3-$H_2O$ 12,08 - 24,80; MAGL: control 10,84 - 28,95; EtOH 16,31 - 42,18; n-3-EtOH 16,34 - 54,29; n-3-$H_2O$ 14,39 - 38,95; NAPE-PLD: control 9,76 - 16,25; EtOH 11,62 – 17,14; n-3-EtOH 8,53 – 12,64; n-3-$H_2O$ 10,63 -8,478; FAAH: control 10,23- 25,93; EtOH 11,59 - 31,29; n-3-EtOH 10,93 - 19,11; n-3-$H_2O$ 10,13 - 20,90; $G\alpha_O$: control 9,60 – 14,71; EtOH 8,92 – 9,20; n-3-EtOH 8,12 – 0,94; n-3-$H_2O$ 6,45 + 9,23; $G\alpha_{i1}$: control 9,77 - 14,79; EtOH 9,63 - 14,78; n-3-EtOH 8,27 - 7.41; n-3-$H_2O$ 8,89 - 9,41; $G\alpha_{i2}$: control 10,95 - 31,61; EtOH 14,02 - 48,74; n-3-EtOH 10,05 - 29,61; n-3-$H_2O$ 11,18 - 32,18; $G\alpha_{i3}$: control 9,29 - 11,49; EtOH 8,72 – 8,90; n-3-EtOH 9,07 + 1148; n-3-$H_2O$ 6,92 - 1,49. The statistical significance of differences between slopes was determined using the extra-sum-of-squares F test (F) method, analyzing the goodness of fit of the experimental data from each experimental groups as a whole (Null hypothesis = Slope same for all data sets) with the goodness of fit of the experimental data for each experimental group individually (Alternative hypothesis = Slope different for each data set).

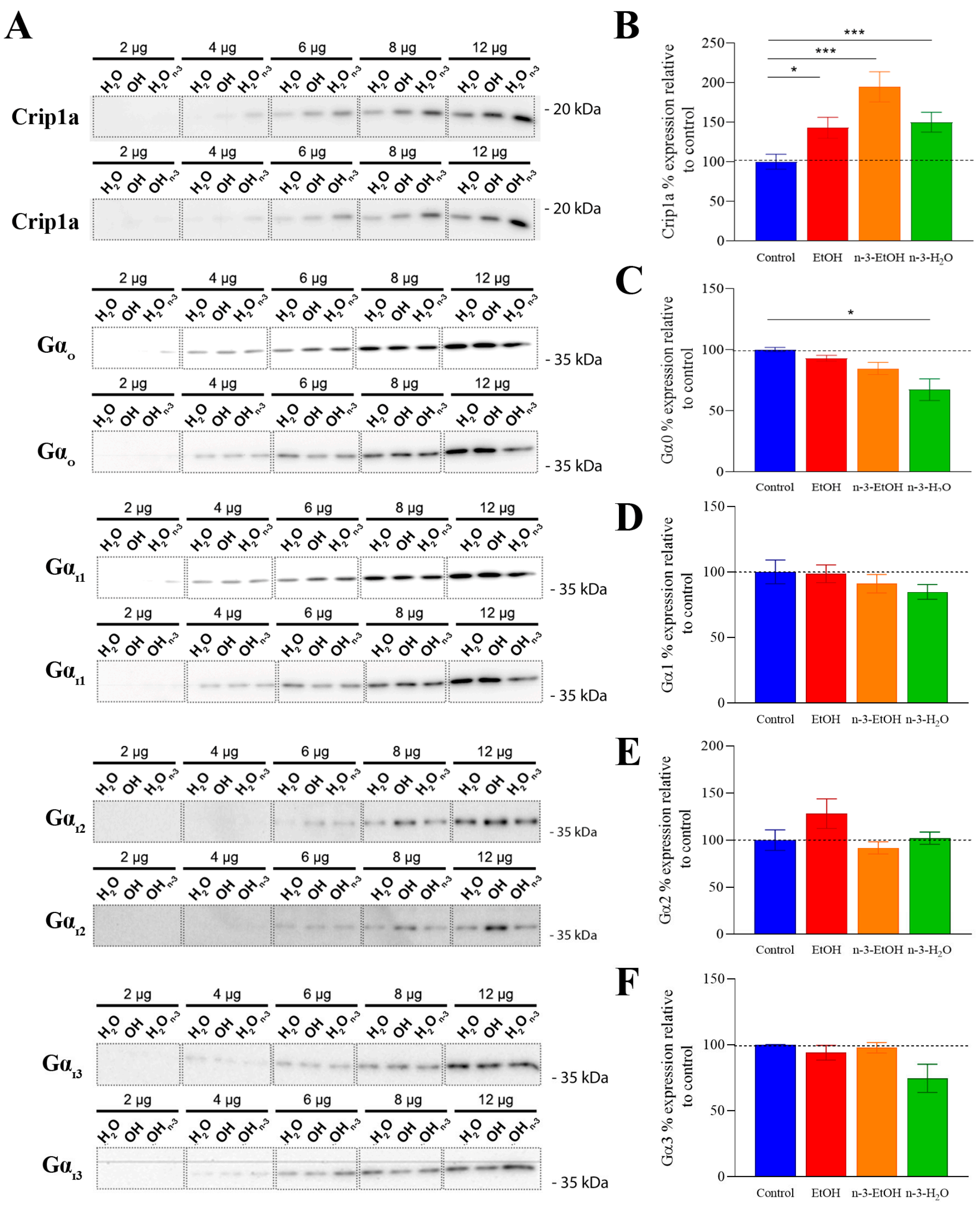


**Supplementary Figure S3. Relative expression of Crip1a and $G\alpha_{i/o}$ subunits in hippocampal synaptosomes from control, EtOH, n-3-EtOH and n-3-$H_2O$ groups. (A)** Representative Western blot analysis with increasing amounts of synaptosomal protein (2, 4, 6, 8, and 12 µg/lane). Protein loading was validated using the Coomassie Brilliant Blue staining. Molecular weights of immunoreactive signals were determined using standard markers (indicated in the figure). Protein migration was consistent with the expected molecular masses (observed weights: $G\alpha_o$, 40.1 kDa; $G\alpha_{i1}$, 40.5 kDa; $G\alpha_{i2}$, 40.4 kDa; $G\alpha_{i3}$, 40.5 kDa; Crip1a, 18.6 kDa). $H_2O$, OH, $H_2O_{n\text{-}3}$ and $OH_{n\text{-}3}$ correspond to control, EtOH, n-3-$H_2O$ and n-3-EtOH groups, respectively. **(B-F)** Histogram of relative expression of Crip1a (EtOH vs. Control p=0.0356; n-3-EtOH vs. Control p=0.0006; n-3-$H_2O$ vs. Control p=0.008), $G\alpha_o$ (n-3-$H_2O$ vs. Control p=0.0378), $G\alpha_{i1}$, $G\alpha_{i2}$, $G\alpha_{i3}$. Linear regression analysis graphs for each protein are provided in Supplementary Figure 2 and were determined using the slope comparison method using an extra sum-of-squares F-test. Significance levels: *p<0.05; **p<0.01; ***p<0.001; ****p<0.0001.

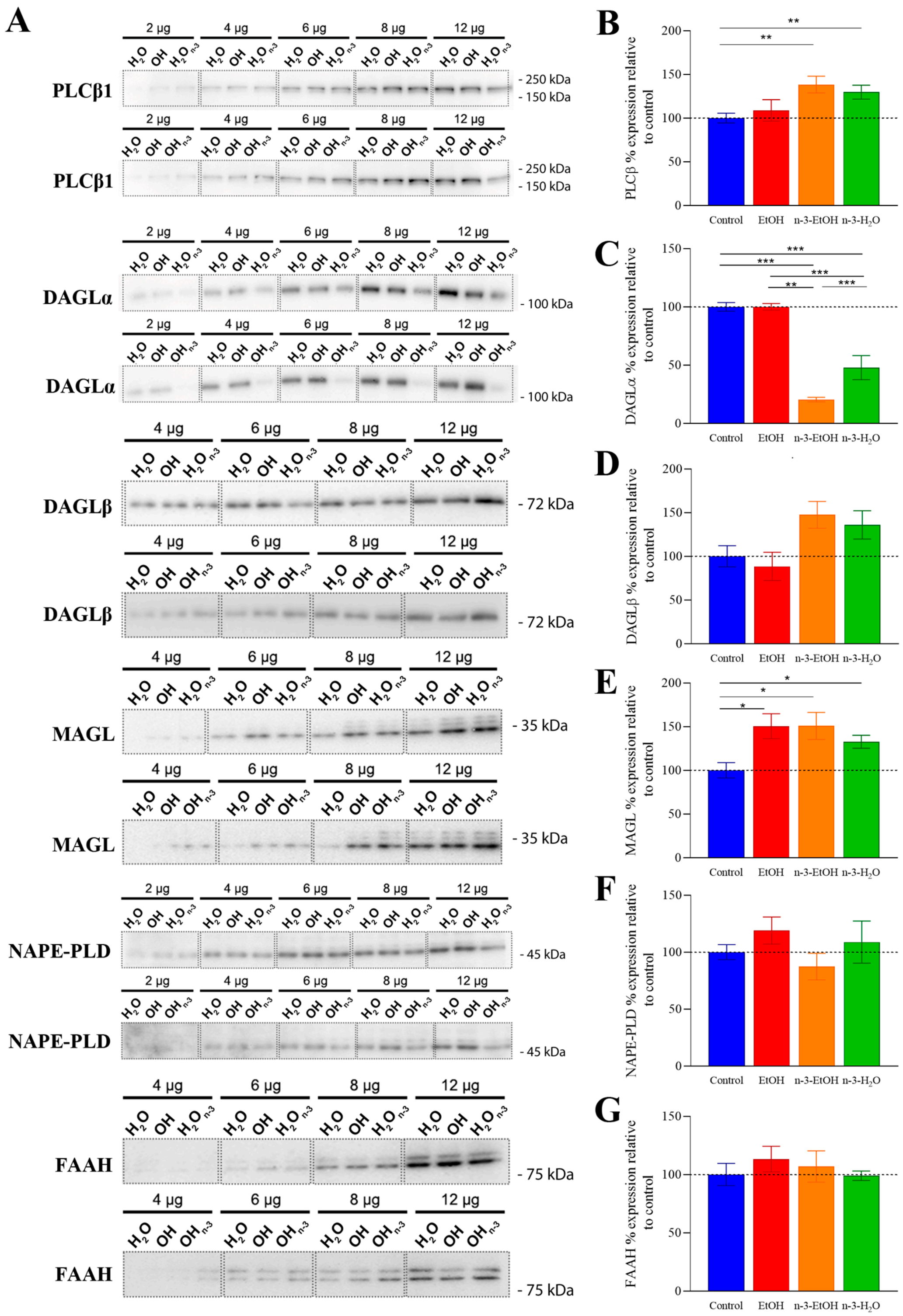


**Supplementary Figure S4. Relative expression of key enzymes involved in AEA and 2-AG synthesis and degradation in hippocampal synaptosomes from control, EtOH, n-3-EtOH and n-3-$H_2O$ groups. (A)** Representative Western blots with increasing amounts of synaptosomal protein (2, 4, 6, 8, and 12 µg per lane). Protein loading was validated using the Coomassie Brilliant Blue gel staining. Molecular weights of the immunoreactive bands were determined with standard markers (as indicated in the figure) and matched the expected molecular masses: (PLCβ1: 138.3 kDa and 133.3 kDa, corresponding to the β1a and β1b isoforms, respectively; DAGLα: 115.3 kDa; DAGLβ: 73.9 kDa; MAGL: 33.3 kDa; NAPE-PLD: 45.8 kDa; FAAH: 63.2 kDa. $H_2O$, OH, $H_2O_{n\text{-}3}$ and $OH_{n\text{-}3}$ correspond to control, EtOH, n-3-$H_2O$ and n-3-EtOH groups, respectively. **(B-G)** Histogram of relative expression PLCβ1 (n-3-EtOH vs. Control $p=0.0042$; n-3-$H_2O$ vs. Control $p=0.009$), DAGLα (n-3-EtOH vs. Control $p=0.0006$; n-3-EtOH vs. EtOH $p=0.0024$; n-3-$H_2O$ vs. Control $p=0.0006$; n-3-$H_2O$ vs. EtOH $p=0.0006$; n-3-$H_2O$ vs. n-3-EtOH $p=0.0024$), DAGLβ, MAGL (EtOH vs. Control $p=0.036$; n-3-EtOH vs. Control $p=0.049$; n-3-$H_2O$ vs. Control $p=0.049$), NAPE-PLD and FAAH. Linear regression

analysis graphs for each protein are provided in Supplementary Figure 2 and were determined using the slope comparison method using an extra sum-of-squares F-test. Significance levels: *p<0.05; **p<0.01; ***p<0.001.

## Supplementary tables

| | Excitatory terminals | | Inhibitory terminals | | Astrocytic processes | |
|---|---|---|---|---|---|---|
| | **% CB1+** | **Density** | **% CB1+** | **Density** | **% CB1+** | **Density** |
| **Control** | 25.75 ± 3.37 | 0.84 ± 0.03 | 91.28 ± 2.20 | 6.20 ± 0.26 | 34.17 ± 2.96 | 0.78 ± 0.03 |
| **EtOH** | 18.92 ± 2.75 | 0.80 ± 0.03 | 83.27 ± 4.64 | 7.58 ± 0.25** | 33.57 ± 2.83 | 0.69 ± 0.03 |
| **n-3-EtOH** | 12.12 ± 2.10* | 1.01 ± 0.06*ΦΦ | 87.18 ± 1.9 | 9.07 ± 0.34****ΦΦ | 26.15 ± 2.20 | 0.70 ± 0.03 |
| **n-3- $H_2O$** | 18.33 ± 2.13 | 0.87 ± 0.04 | 90.78 ± 1.63 | 6.83 ± 0.26†††† | 28.13 ± 2.33 | 0.66 ± 0.03 |
| *DID* | p=0.0174* | ns | p=0.0486* | p<0.0001**** | ns | ns |
| *Diet* | p=0.0099** | p=0.0011** | ns | p=0.0002*** | p=0.0130* | ns |
| *Interaction* | ns | p=0.0170* | ns | ns | ns | ns |

**Supplementary Table S1. Density and percentage of CB1 receptor-positive excitatory and inhibitory terminals and GLAST-stained astrocytes in the middle third of the DG of control, EtOH, n-3-EtOH and n-3-$H_2O$ groups** (n= 3 mice per group). Values are expressed as mean ± SEM. The statistical significance was analyzed by two-way ANOVA and Tukey's multiple comparison test: *p<0.05, **p<0.01, ****p<0.0001 compared to control; ΦΦp<0.01 when compared to EtOH; and ††††p<0.0001 compared to n-3-EtOH. Two way ANOVA examining the effects of DID, diet, and their interaction: ns= no significance, *p<0.05, **p<0.01, ***p<0.001, ****p<0.0001.

| | **WIN 55,212-2** | **Capsaicin** | **Capsaicin +AMG9810** |
|---|---|---|---|
| **Control** | 65.71 ±0.61* | | |
| **EtOH** | 105.80 ±0.35 | | |
| **n-3-EtOH** | 102.60 ±0.41 | 136.00 ±0.49**** | 105.20 ±0.91 |
| **n-3-$H_2O$** | 139.50 ±0.35* | | |

**Supplementary Table S2. Excitatory synaptic transmission at MPP-granule cell synapses in the four experimental conditions.** WIN 55,212-2 (5 μM) (Control: response vs. baseline p=0.0373; n-3-$H_2O$: response vs. baseline p=0.0361), Capsaicin (1 μM) (n-3-EtOH: response vs. baseline p<0.0001), Capsaicin (1 μM) + AMG9810 (3 μM) were used. Data are expressed as mean ± SEM of at least three different mice. Parametric or non-parametric test (Paired t-test or Wilcoxon matched-pairs signed rank test, respectively) for drugs vs. baseline) were used. *p<0.05, **p<0.01, ****p<0.0001 vs. baseline.

| | | | |
|---|---|---|---|
| **AMG9810** (3 μM) | 108.80 ±2.06** | **THL** (10 μM) | 142.70 ± 0.83Φ |
| **AM251** (4 μM) | 131.80 ± 0.31Φ | **LEI401** (10 μM) | 109.00 ± 0.33** |
| **Latrunculin A** (500 μM) | 133.10 ± 1.05ΦΦ | **AM404** (30 μM) | 100.50 ± 1.98**** |
| **D-AP5** (50 μM) | 102.00 ± 1.72**** | **URB597** (2 μM) | 108.50 ± 0.85** |
| **ω-conotoxin GVIA** (1 μM) | 139.70 ± 0.33Φ | | |

**Supplementary Table S3. Excitatory synaptic plasticity at MPP-granule cell synapses in the four experimental conditions.** Low-frequency stimulation (10 Hz for 10 minutes) was applied to investigate the mechanisms underlying MPP-LTP in the n-3-EtOH group, and fEPSPs were compared in the presence or absence of various compounds. Data are expressed as mean ± SEM of at least three different animals. Dunn's test was used **p<0.01, ****p<0.0001 (response *vs*. LTP in

absence of compound) and paired t-test or Wilcoxon matched-pairs signed rank test were used ᶲp<0.05, ᶲᶲp<0.01 (response *vs*. baseline).

| | $H_2O$ | EtOH | n-3-EtOH | n-3-$H_2O$ |
|---|---|---|---|---|
| **DAY 1** | 68.89 ± 5.55 | 129.40 ± 13.35** | 87.58 ± 11.45 | 93.00 ± 15.55 |
| **DAY 2** | 33.85 ± 4.12 | 72.62 ± 13.93 | 37.38 ± 7.78 | 63.10 ± 15.82 |
| **DAY 3** | 21.65 ± 3.33 | 62.18 ± 13.59* | 26.10 ± 5.42 | 34.60 ± 9.07 |
| **DAY 4** | 19.83 ± 3.71 | 39.60 ± 13.12 | 24.85 ± 9.53 | 31.18 ± 8.89 |
| **DAY 5** | 11.78 ± 2.13 | 35.24 ± 11.45 | 14.85 ± 3.51 | 18.80 ± 6.49 |

**Supplementary Table S4. Barnes Maze performance of the control (n=18), EtOH (n=17), n-3-EtOH (n=10) and n-3-$H_2O$ (n=10) groups.** Time required to locate the escape box over five days of testing Day 1: EtOH vs. Control p=0.0025; Day 3: EtOH vs. Control p=0.0414). Data are presented as mean ± SEM and analyzed using two-way ANOVA followed by Tukey's multiple comparison test. Significance levels: *p<0.05, **p < 0.01 compared to control. Two way ANOVA revealed time effect ****p<0.0001 and experimental group effect *p<0.05, but no interaction between them p>0.05.

| | $H_2O$ | EtOH | n-3-EtOH | n-3-$H_2O$ |
|---|---|---|---|---|
| **DAY 1** | 15.49 ± 1.23 | 25.16 ± 2.66* | 17.15 ± 2.89 | 16.58 ± 2.36 |
| **DAY 2** | 11.22 ± 1.42 | 19.13 ± 3.66 | 9.55 ± 2.38 | 11.83 ± 2.40 |
| **DAY 3** | 9.14 ± 1.25 | 17.93 ± 2.69* | 9.45 ± 2.46 | 11.30 ± 2.83 |
| **DAY 4** | 8.46 ± 1.29 | 12.91 ± 2.91 | 8.73 ± 3.77 | 10.83 ± 3.29 |
| **DAY 5** | 4.50 ± 0.98 | 10.44 ± 2.68 | 4.65 ± 1.15 | 6.53 ± 2.37 |

**Supplementary Table S5. Barnes Maze performance of the control (n=18), EtOH (n=17), n-3-EtOH (n=10) and n-3-$H_2O$ (n=10) groups.** Number of errors made to locate the escape box over five days of testing (Day 1: EtOH vs. Control p=0.0217; Day 3: EtOH vs. Control p=0.0300). Data are presented as mean ± SEM and analyzed using two-way ANOVA followed by Tukey's multiple comparison test. Significance levels: *p<0.05 compared to control. Two way ANOVA revealed time ****p<0.0001 and experimental group effect ****p<0.0001, but no interaction between them p>0.05.

| Antigen | [µg/mL] | Species (clonality) | Immunogen | Source, Cat. |
|---|---|---|---|---|
| **CB1** | 2 | Guinea pig (polyclonal) | Mouse CB1, C-terminal 31 aa. | Nittobo Medical Co., CB1-GP-Af530 |
| **GLAST** | 0.3 | Rabbit (polyclonal) | Mouse GLAST, C-terminal 41 aa | Nittobo Medical Co., GLAST-Rb-Af660 |
| **Guinea pig IgG** | 0.8 | Goat (polyclonal). 1.4 nm gold-conjugated | | Nanoprobes, #2055 |
| **Rabbit IgG** | 7.5 | Goat (polyclonal). Biotinylated | | Vector Labs, BA-1000 |

**Supplementary Table S6. Primary and secondary antibodies used in immunoelectron microscopy.**

| | Control | | | | EtOH | | | | n-3-EtOH | | | | n-3- $H_2O$ | | | |
|---|---|---|---|---|---|---|---|---|---|---|---|---|---|---|---|---|
| | n 1 | n 2 | n 3 | **Total** | n 1 | n 2 | n 3 | **Total** | n 1 | n 2 | n 3 | **Total** | n 1 | n 2 | n 3 | **Total** |
| **Excit. Ter.** | 289 | 247 | 395 | **931** | 394 | 288 | 323 | **1005** | 287 | 255 | 388 | **980** | 372 | 253 | 355 | **930** |
| **Inhib. Ter.** | 55 | 76 | 58 | **189** | 67 | 70 | 62 | **199** | 54 | 76 | 64 | **200** | 58 | 64 | 78 | **194** |
| **Astr. Proc.** | 365 | 305 | 213 | **883** | 374 | 216 | 269 | **859** | 384 | 322 | 348 | **921** | 363 | 264 | 294 | **1054** |

**Supplementary Table S7. Counts of excitatory and inhibitory terminals and GLAST-stained astrocytic processes in the four experimental groups.**

| Antigen | Dilution | Species and clonality | Isotype | Immunogen | Source, Cat. |
|---|---|---|---|---|---|
| **CB1** | 1:1000 | Goat polyclonal | Serum | Mouse CB1, C-terminal 31 aa (NM007726) | Nittobo Medical Co., CB1-Go-Af450 |
| **NAPE-PLD** | 1:1000 | Guinea pig polyclonal | Serum | Mouse N-terminal 1-41aa (AB112350) | Nittobo Medical Co., NAPE-PLD-GP-Af720 |
| **FAAH** | 1:1000 | Rabbit polyclonal | Not specified | Synthetic peptide from the C-terminal region of rat FAAH | Cayman chemical, 101600 |
| **Crip1a** | 1:500 | Rabbit polyclonal | IgG | Peptide mapping within an internal region of Crip1 of human origin | Santa Cruz Biotech., sc-137401 |
| **PLCβ1** | 1:2000 | Sheep Polyclonal | IgG | Recombinant human PLC-β1, residues 27-245 | Novus Biologicals, AF4466 |
| **DAGL$\alpha$** | 1:1000 | Rabbit polyclonal | Serum | Mouse DAGL$\alpha$, C-terminal 42 aa (NM198114) | Nittobo Medical Co., DGLa-Rb-Af380 |
| **DAGLβ** | 1:2000 | Rabbit monoclonal | IgG | Peptide corresponding to residues surrounding Leu505 of human DAGLβ | Cell signalling, 12574 |
| **MAGL** | 1:1000 | Rabbit polyclonal | Serum | Mouse MAGL, residues 1-35 | Nittobo Medical Co., MGL-Rb-Af200 |
| **$G\alpha_o$** | 1:200 | Mouse monoclonal | $IgG_1$ kappa light chain | $G\alpha_o$ of bovine origin | Santa Cruz Biotech., sc-13532 |
| **$G\alpha_{i1}$** | 1:200 | Mouse monoclonal | $IgG_{2b}$ kappa light chain | $G\alpha_{i1}$ of rat origin | Santa Cruz Biotech., sc-13533 |
| **$G\alpha_{i2}$** | 1:200 | Mouse monoclonal | $IgG_{2b}$ kappa light chain | $G\alpha_{i2}$ of rat origin | Santa Cruz Biotech., sc-13534 |
| **$G\alpha_{i3}$** | 1:200 | Mouse monoclonal | $IgG_3$ kappa light chain | Epitope mapping between residues 339-354 of $G\alpha_{i3}$ of rat origin | Santa Cruz Biotech., sc-365422 |

**Supplementary Table S8. Primary antibodies used in Western blotting.**
